# Spin-orbit torque manipulation of sub-terahertz magnons in antiferromagnetic *α*-Fe_2_O_3_

**DOI:** 10.1038/s41467-024-48431-w

**Published:** 2024-05-14

**Authors:** Dongsheng Yang, Taeheon Kim, Kyusup Lee, Chang Xu, Yakun Liu, Fei Wang, Shishun Zhao, Dushyant Kumar, Hyunsoo Yang

**Affiliations:** 1 Department of Electrical and Computer Engineering, National University of Singapore, Singapore, Singapore; 2 Electro-Medical Device Research Centre, Korea Electrotechnology Research Institute, Ansan, Republic of Korea

**Keywords:** Spintronics, Magnetic properties and materials

## Abstract

The ability to electrically manipulate antiferromagnetic magnons, essential for extending the operating speed of spintronic devices into the terahertz regime, remains a major challenge. This is because antiferromagnetic magnetism is challenging to perturb using traditional methods such as magnetic fields. Recent developments in spin-orbit torques have opened a possibility of accessing antiferromagnetic magnetic order parameters and controlling terahertz magnons, which has not been experimentally realised yet. Here, we demonstrate the electrical manipulation of sub-terahertz magnons in the *α*-Fe_2_O_3_/Pt antiferromagnetic heterostructure. By applying the spin-orbit torques in the heterostructure, we can modify the magnon dispersion and decrease the magnon frequency in *α*-Fe_2_O_3_, as detected by time-resolved magneto-optical techniques. We have found that optimal tuning occurs when the Néel vector is perpendicular to the injected spin polarisation. Our results represent a significant step towards the development of electrically tunable terahertz spintronic devices.

## Abstract (web summary)

Antiferromagnetic spintronics offer high speed operations, and reduced issues with stray fields compared to ferromagnetic systems, however, antiferromagnets are typically more challenging to manipulate electrically. Here, Yang, Kim, and coauthors demonstrate electrical control of magnon dispersion and frequency in an α-Fe_2_O_3_/Pt heterostructure.

## Introduction

Magnons, the quanta of collective spin-wave excitations, carry the spin angular momentum without moving charges^1,2^, and could serve as potential information carriers, allowing Joule-heat-free data transfer, These features have made them of great interest in the energy-efficient information technology^3–5^. Antiferromagnets, where two-spin sublattices are aligned antiparallel due to the negative exchange interaction, host antiferromagnetic (AFM) magnons which have frequencies that are 2–3 orders of magnitude higher than their ferromagnetic counterparts due to a stronger AFM exchange energy^6,7^, and insulating antiferromagnets have low magnetic dissipation^8^. These features have rendered insulating antiferromagnets as a promising platform for energy-efficient terahertz (THz) magnonics and optoelectronics.

Such devices require active electronic control of THz magnons. One such approach is to utilise current-induced spin-orbit torques (SOT)^9,10^. However, attention has principally been confined to manipulating the static magnetic state, leading to AFM magnetic reorientation/switching^11–14^. In stark contrast, the direct control of the THz spin dynamics in antiferromagnets, such as the magnon frequency, is critical for novel applications such as THz spin nano-oscillators^15–17^ and rectifiers^18–21^. Pioneering efforts to control the magnon frequency have focused on conventional ferromagnetic and ferrimagnetic thin films and two-dimensional exfoliated flakes, whose magnon frequencies reside in tens of GHz^22–28^. Although theoretical works^15,17,29,30^ pointed out that the frequency of the uniform resonant mode (wavevector *k* = 0 magnon) in antiferromagnets can be controlled by SOT, the experimental demonstration, including propagating magnons (*k* ≠ 0), has remained elusive.

**Fig. 1:**
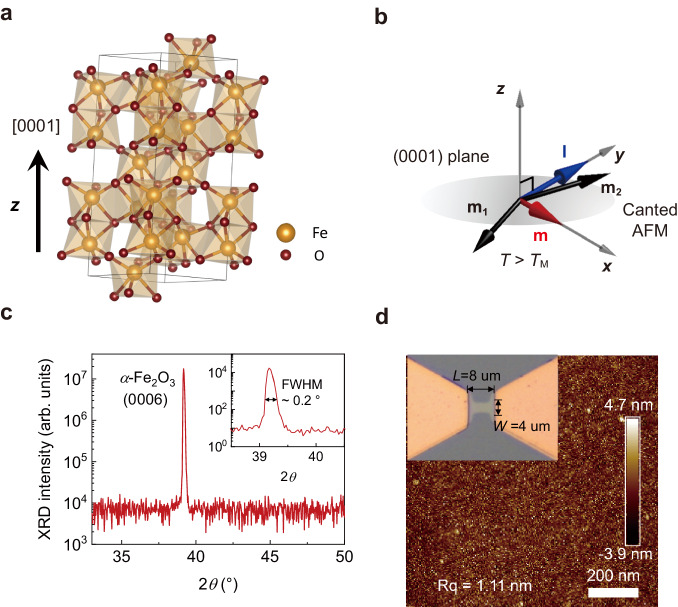
Material characterisation of *α*-Fe_2_O_3_ sample. **a**, **b** Crystal (**a**) and magnetic (**b**) structure of *α*-Fe_2_O_3_ above the Morin temperature *T*_M_ = 261 K. **m,** **l,** **m**_**1**_ and **m**_**2**_ represent a canted moment, Néel vector, and 1st and 2nd sublattice moment. **c** X-ray diffraction (XRD) measurement of the *α*-Fe_2_O_3_ sample. The peak at 39.2° matches well with the position of the *α*-Fe_2_O_3_ (0006) peak. Inset: the zoomed view of the *α*-Fe_2_O_3_ (0006) peak showing the full width at half maximum (FWHM). **d** Atomic force microscopy image of the *α*-Fe_2_O_3_ (0001) sample. The root mean square average (Rq) of the profile heights is 1.11 nm. Inset: the device image with a dimension of 4 μm in width (*W*) and 8 μm in length (*L*).

**Fig. 2:**
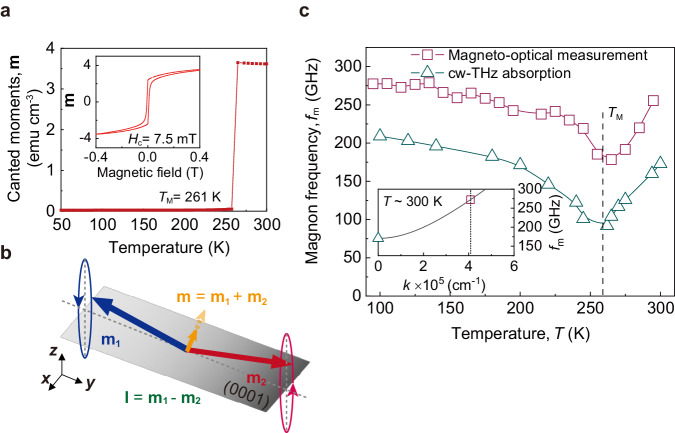
Temperature dependence of *α*-Fe_2_O_3_ magnon frequency *f*_m_ without applying currents. **a** Canted moments **m** as a function of temperature from 50 and 300 K, showing a Morin transition at *T*_M_ ~ 261 K. Inset: the magnetic hysteresis loop with an in-plane coercivity *H*_c_ = 7.5 mT at *T* = 300 K. **b** Configuration of q-AFM spin resonance. Both **l** and **m** are located in the (0001) plane and perpendicular to each other. **c** Temperature dependences of *f*_m_ (wavevector *k* > 0) obtained by magneto-optical measurement (squares) and *f*_m_ (*k* = 0) by continuous-wave terahertz (cw-THz) absorption measurement (triangles). The overall frequency blueshift measured by magneto-optical measurements is evidence of finite *k* magnons. Inset: the calculated *α*-Fe_2_O_3_ magnon dispersion relation for the q-AFM mode along the [0001] direction, in which both *f*_m_ and *k* match well with two measurement results.

Here, we demonstrate the electrical control of the sub-THz magnon frequency in an easy-plane AFM insulator, *α*-Fe_2_O_3_. For this work, we use 5-mm-thick, (0001)-oriented *α*-Fe_2_O_3_ crystals (Fig. 1a, b). Figure 1c, d show the X-ray diffraction pattern and atomic force microscopy image of the *α*-Fe_2_O_3_ sample, respectively, confirming the high crystal quality and the [0001] crystallographic orientation. The magnetisation versus temperature is displayed in Fig. 2a. The magnetic phase transition in *α*-Fe_2_O_3_, called the Morin transition, occurs at the Morin temperature *T*_M_, which is ~261 K. The magnetic configuration above *T*_M_ is schematically illustrated in Fig. 1b. The magnetic order of two sublattices, **m**_1_ and **m**_2_, is nearly antiparallel but slightly canted due to the Dzyaloshinskii-Moriya interaction (DMI)^31^, resulting in a nearly unit-length Néel vector **l** = (**m**_2_ − **m**_1_)/2 and a perpendicular canted magnetic moment **m** = (**m**_1_ + **m**_2_)/2 and on the (0001) plane. The small coercivity around 7.5 mT (the inset of Fig. 2a) results from the insignificant in-plane magneto-anisotropy of *α*-Fe_2_O_3_. As a result, a weak external magnetic field (**H**) could align the direction of **m**, leading to a magnetic configuration with **l** ⊥ **H** and **m** // **H** in the (0001) plane. At room temperature, two eigenmodes of magnon in *α*-Fe_2_O_3_ correspond to the out-of-plane and in-plane elliptical precessional motion of **m**_1_ and **m**_2_ lying in the (0001) plane^32^. Our study focuses on the out-of-plane precessional mode, also known as the quasi-antiferromagnetic (q-AFM) mode, which exhibits sub-THz magnon frequencies (Fig. 2b)^33^. In contrast, the frequency of the in-plane mode, or the quasi-ferromagnetic (q-FM) mode, resides in the range of several GHz in the absence of **H**^34,35^.

## Results

### Sub-THz magnon measurements in *α*-Fe_2_O_3_ with currents

In this work, we use the time-resolved magneto-optical method for observing the sub-THz magnons in *α*-Fe_2_O_3_ (Methods, Supplementary Note 1). This method is a benchmark technique to investigate the spin dynamics in AFM insulators through magneto-optical interactions, i.e., the Faraday effect and Cotton-Mouton effect. Specifically, the spin dynamics in *α*-Fe_2_O_3_ are obtained via ultrafast excitation of the pump pulse with the photon energy of 3.1 eV above the *α*-Fe_2_O_3_ bandgap and detected by tracking the polarisation rotation of the probe pulse using typical magneto-optical detection, similar to previous experiments^36–39^ (Supplementary Notes 2 and 3). We adopt this method as it is sensitive to the interfacial effect where SOT tuning prevails and can measure higher-frequency magnon components with finite *k*^26,40^.

To identify the value of magnon wavevector *k* measured by the time-resolved magneto-optical method, we first measure the resonance (*k* = 0 magnon) frequency by using a continuous-wave THz (cw-THz) absorption method (Supplementary Note 4)^41^. The temperature-dependent q-AFM magnon frequency *f*_m_ of *α*-Fe_2_O_3_ without the current using both methods are summarised in Fig. 2c. The results of cw-THz absorption method indicate a resonance frequency of approximately 200 GHz at room temperature with a dip in the vicinity of the *T*_M_, which is consistent with the q-AFM resonance mode (*k* = 0 magnon) in *α*-Fe_2_O_3_^33^. In comparison, the *f*_m_ measured by the time-resolved magneto-optical method displays a similar trend but is consistently shifted by ~70 GHz over that by the cw-THz absorption method. This consistent blueshift in frequency constitutes concrete evidence for the existence of finite *k* magnons, similar to the previous report on AFM DyFeO_3_^40^. In this case, the value of probed *k* can be determined using the Bragg equation^42^ *k* = 2*k*_0_*n*(*λ*_0_)cos*δ‘* in which *k*_0_ is the wavenumber of the probe light, *n*(*λ*_0_) is the refractive index of *α*-Fe_2_O_3_/Pt heterostructure at the centre wavelength of the probe beam *λ*_0_ = 800 nm and *δ‘* is the refraction angle of the probe beam at an incidence angle *δ*. By applying our experimental parameters^43^ with *n*(*λ*_0_) equating 2.6 at *λ*_0_ = 800 nm and *δ* being 0°, the measured *k* in the time-resolved magneto-optical experiment is estimated to be 4.08 × 10^5 ^cm^−1^ (Supplementary Note 5).

**Fig. 3:**
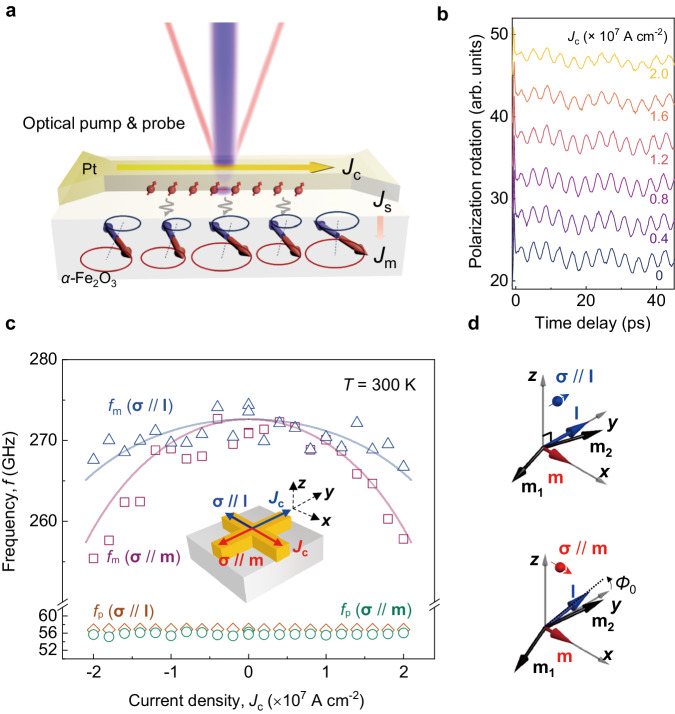
Experimental observation of SOT-manipulated q-AFM *f*_m_ of *α*-Fe_2_O_3_ at *T* = 300 K. **a** Schematics of the SOT-tuned AFM magnon measurement using the time-resolved magneto-optical method (Methods). Coherent magnons in *α*-Fe_2_O_3_ are excited via femtosecond optical pumping and manifest the polarisation rotation of the probe beam via magneto-optical interaction. *J*_c_, *J*_s_, and *J*_m_ are the injected charge current, spin current and magnon current, respectively. **b** *f*_m_ is manipulated by *J*_s_ injected from the adjacent Pt layer and detected as the oscillation signal using a balanced detection scheme (**σ** // **m** configuration). **σ**: spin polarisation. The overlapped phonon oscillation at phonon frequency *f*_p_ = 55 GHz can be used to judge SOT tuning. **c** Summary of SOT-tuned *f*_m_ and *f*_p_ with *J*_c_ at both **σ** // **l** and **σ** // **m** configurations. *f*_m_ at **σ** // **l** (**σ** // **m**) is denoted by triangles (squares) and *f*_p_ at **σ** // **l** (**σ** // **m**) is denoted by diamonds (circles). The solid lines represent the theoretical calculation results (Supplementary Note 7). **d** SOT-manipulated spin configurations at **σ** // **l** and **σ** // **m**, where *Φ*_0_ is SOT-induced reorientation of **l**.

To quantitatively correlate the *f*_m_ with different *k*, the magnon dispersion of *α*-Fe_2_O_3_ along [0001] or the *z* direction (Supplementary Notes 6 and 7) is given by

1 $${f}_{{{{{{\rm{m}}}}}}}(k,{J}_{{{{{{\rm{c}}}}}}})=\sqrt{{({\upsilon }_{0}k)}^{2}+{f}_{{{{{{\rm{m}}}}}},{{{{{\rm{k}}}}}}=0}^{2}({J}_{{{{{{\rm{c}}}}}}})},$$

where υ_0_ ~ 32 km s^−1^ is the limiting magnon velocity along the [0001] crystalline direction^44^, ${f}_{{{{{{\rm{m}}}}}},{{{{{\rm{k}}}}}}=0}$ is the q-AFM magnon frequency at *k* = 0, and *J*_c_ is the applied charge current. The value of *f*_m_ at *k* = 4.08 × 10^5 ^cm^−1^ is larger than that of ${f}_{{{{{{\rm{m}}}}}},{{{{{\rm{k}}}}}}=0}$ as it is shifted by υ_0_*k*. Here, *λ*_0_ is dominant in determining *k*, whereas the *δ* dependence on *k* is small; *δ* = 0° (*δ* = 30°) corresponds to *k* = 4.08 ×10^5 ^cm^−1^ (*k* = 4 × 10^5 ^cm^−1^) and *f*_m_ = 272 GHz (*f*_m_ = 269 GHz). As shown in the inset of Fig. 2c, both *f*_m_ and *k* extracted from both time-resolved magneto-optical and cw-THz absorption methods agree well with the calculated magnon dispersion, as a cross-check of the *k* value extracted by the Bragg equation. In addition, we observe a slow oscillation mode at ~55 GHz (Fig. 3). This corresponds to a laser-induced acoustic phonon mode, which has been widely studied previously^40,42^ (Supplementary Note 8).

### SOT manipulation of sub-THz *α*-Fe_2_O_3_ magnons

Next, we explore the SOT manipulation for the *α*-Fe_2_O_3_ magnons at *k* = 4.08 × 10^5 ^cm^−1^. As illustrated in Fig. 3a, the adjacent Pt is a strong spin-orbit coupling material and acts as the spin source. By applying *J*_c_ across the Pt layer, transverse spin accumulations are generated at the *α*-Fe_2_O_3_/Pt interface with the spin polarisation **σ**. The spin accumulation induces a SOT on the magnetic order of *α*-Fe_2_O_3_ and generates magnon current density *J*_m_, allowing for the manipulating of *f*_m_. To understand the modified magnon dispersion, we adopt a magnon-mediated spin current *J*_m_, which can propagate into *α*-Fe_2_O_3_ (Method and Supplementary Note 9). By monitoring the polarisation rotation of the reflected probe pulses under different *J*_c_, the manipulation of the *f*_m_ can be studied as a function of *J*_c_ (Fig. 3b, c).

To confirm the dominant mechanism of SOT rather than other unwanted effects (e.g., heating and strain), we perform the measurements under both **σ** // **m** and **σ** // **l** configurations. Due to the large size of the magnetic domain in the *α*-Fe_2_O_3_ sample, which exceeds hundreds of micrometres, our device can be considered in a saturated mono-domain condition (Methods and Supplementary Fig. 13). Figure 3c, d summarises the *f*_m_ with different *J*_c_ and under both configurations (**σ** // **l** and **σ** // **m**). The direction of **m** in *α*-Fe_2_O_3_ is aligned along the external magnetic field **H** and is orthogonal to the orientation of **l** in the (0001) plane, as confirmed by spin Hall magnetoresistance measurements (Supplementary Fig. 14). No magnetic field is applied during the time-resolved magneto-optical measurement.

At the **σ** // **m** configuration, we observe that *f*_m_ decreases nonlinearly with increasing *J*_c_ from 272 GHz at *J*_c_ = 0 A cm^−2^ to 258 GHz at *J*_c_ = 2 × 10^7 ^A cm^−2^, resulting in a *f*_m_ tuning of −14 GHz (the minus sign refers to a redshift of *f*_m_). We note that this redshift of *f*_m_ is opposite to the heating-induced effect that increases *f*_m_ when *T* > *T*_M_. Moreover, we find that the tuning effect is symmetrical with the polarity of *J*_c_, which is distinct from that of ferromagnets^26^. This is reasonable as *J*_c_ does not break the symmetry of **l** in *α*-Fe_2_O_3_^8^. Furthermore, we find that the frequency of the acoustic phonon *f*_p_ remains unchanged with *J*_c_, which supports that the tuning of *f*_m_ is due to spin-related mechanisms rather than the strain resulting from the current flow. We further developed a comprehensive spin-wave model that combined the SOT and AFM magnon dispersion to explain our experimental observations (Supplementary Notes 7 and 8). In the **σ** // **m** configuration, SOT reorients **l** from the equilibrium axis (the *y*-axis in Fig. 3d), reducing the effective anisotropy and finally resulting in the redshift of *f*_m_. As shown in Fig. 3c, the simulation result in a solid line matches well with both the polarity dependence and the amount of *f*_m_ change.

We then study the second configuration with **σ** // **l**. As shown in Fig. 3c, we observe a similar redshifted trend in *f*_m_ by increasing the current density, with a reduced amount of −6 GHz tuning at *J*_c_ = 2 × 10^7 ^A cm^−2^, ~43% of that at **σ** // **m**. Therefore, *f*_m_ reveals distinct dependences on the configuration, showing that the direction of **l** with respect to **σ** is important for efficient *f*_m_ tuning. This anisotropic feature is analogous to the previous report on the *α*-Fe_2_O_3_ spin transport, where spins propagate longer with **σ** // **l** than at **σ** // **m**^8^. Our result indicates that SOT is less pronounced at **σ** // **l** than at **σ** // **m**, leading to a reduced *f*_m_ tuning. Furthermore, the simulated *f*_m_ at **σ** // **l** in Fig. 3c matches the experimental results.

## Discussion

**Fig. 4:**
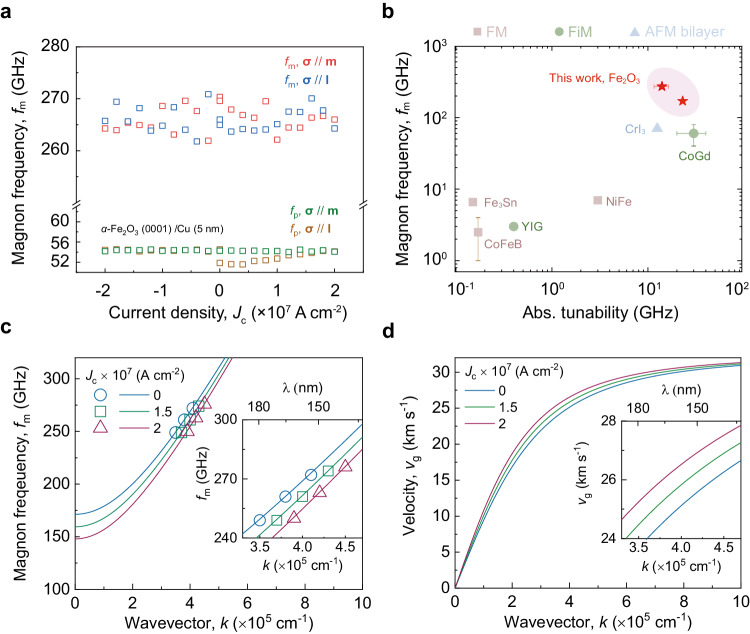
Control measurement of *α*-Fe_2_O_3_/Cu (5 nm) and simulation results of SOT-tuned *α*-Fe_2_O_3_ magnon dispersion. **a** Current-dependence of *f*_m_ and *f*_p_ in the controlled *α*-Fe_2_O_3_ (0001)/Cu (5 nm) device under two-spin configurations. Neither *f*_m_ nor *f*_p_ is tuned by *J*_c_ at both spin configurations, which suggests that SOT is essential for the observed *f*_m_ tuning in Fig. 3. **b** Comparison of the tunability and *f*_m_ by electrical means summarised in Supplementary Table 1. Two red stars represent *f*_m_ our tuning results for *k* = 0 and *k* = 4.08 × 10^5 ^cm^-1^. The error bars indicate the uncertainty of the data extracted from references. FM: ferromagnet, FiM: Ferrimagnet, AFM: antiferromagnet. **c** Numerical (symbols) and analytical (lines) calculated magnon dispersion in the **σ** // **m** configuration. Maximum *f*_m_ tunability of −23 GHz occurs for the zone centre (resonant) mode at *J*_c_ = 2 × 10^7 ^A cm^-2^. **d** Magnon group velocity with *k* extracted from **c**. The magnon group velocity increases effectively for intermediate *k*, leading to an increase from 25.3 to 26.7 km s^-1^ at *J*_c_ = 2 × 10^7 ^A cm^-2^ for *k* = 4.08 × 10^5 ^cm^-1^.

To further confirm the role of SOT on the change in *f*_m_, we conduct a control measurement in the *α*-Fe_2_O_3_/Cu (5 nm) device, where we have used Cu, due to its negligible spin-orbit coupling strength. No change is observed in *f*_m_ at either **σ** // **m** or **σ** // **l** configuration (Fig. 4a). These results confirm the SOT origin of our observed *f*_m_ tuning in Fig. 3c and rule out other strain and heating effects. Next, we consider the current-induced heating effect. The limited temperature rise may slightly increase *f*_m_ at *T* > *T*_M_ (Fig. 2c) but cannot account for the observed decrease of *f*_m_ in Fig. 3c (Supplementary Note 10). Therefore, we conclude that SOT dominates the magnon tuning in our experiments. Figure 4b summarises the tunability and *f*_m_ in various magnetic materials by electrical means (Supplementary Table 1 for details). The SOT effect on the spin lifetime in Supplementary Note 11 shows no change for both spin configurations as the applied current density is smaller than that for self-oscillation.

Noticeably, tuning *f*_m_ using SOT is significantly more energy efficient compared to current-induced Oersted fields, *H*_Oe_. The induced *H*_Oe_, by Ampère’s law (${H}_{{{{{{\rm{Oe}}}}}}} \sim {\mu }_{0}{J}_{{{{{{\rm{C}}}}}}}{t}_{{{{{{\rm{Pt}}}}}}}/2$) is as small as *H*_oe_ = 0.63 mT for *J*_c_ = 2 × 10^7 ^A cm^-2^, where *μ*_0_ is the vacuum permeability and *t*_Pt_ is the thickness of Pt layer. The *f*_m_ change induced by *H*_Oe_ is expected to be *γH*_Oe_/2*π* $\approx$ 0.017 GHz, where *γ* is the electron gyromagnetic ratio, which is nearly 10^3^ times smaller in magnitude than the manipulation of *f*_m_ achieved by SOT.

We calculate the magnon dispersion relation at **σ** // **m** as a function of *J*_c_ and plot in Fig. 4c (Supplementary Notes 6 and 7). As *J*_c_ increases, the dispersion relation curves near the *k* = 0 are redshifted downward. However, the amount of frequency shift at large *k* is smaller. Therefore, maximised frequency tuning occurs for the resonant mode (*k* = 0), resulting in a frequency tunability of −24 GHz/(2 × 10^7 ^A cm^-2^). The SOT increases the band curvature and thus increases the magnon group velocity, *υ*_g_ = $2\pi \times \partial {f}_{m}/\partial k$. As shown in Fig. 4d, for *k* = 4.08 × 10^5 ^cm^-1^ magnons, *υ*_g_ increases from 25.3 to 26.7 km s^-1^ at *J*_c_ = 2 × 10^7 ^A cm^-2^ with **σ** // **m**. Furthermore, we explore the SOT-induced *f*_m_ modulation with varying the exchange field *H*_E_ and effective magnetic anisotropy field *H*_eff,K_ for **σ** // **m** (Supplementary Note 12). In this case, antiferromagnets with a high *H*_E_ and low *H*_eff,K_ are preferred for efficient *f*_m_ tuning, which is in line with the theoretical result that IrMn with a much higher *H*_eff,K_ ~ 5000 Oe yields a reduced tuning efficiency^45^.

As shown in Fig. 4b, our work marks the electrically manipulated *f*_m_ at the highest frequency (270 GHz for *k* = 4.08 × 10^5 ^cm^-1^ and 170 GHz for *k* = 0) ever reported. For magnetic materials with various values of *f*_m_, it is fair to compare their tuning efficiency, defined as *η*(*J*_c_) = [*f*_m_(*J*_c_) – *f*_m_(0)]*/*[*J*_c_ × *f*_m_(*J*_c_)], under a normalised input amplitude. The efficiency of our *α*-Fe_2_O_3_/Pt heterostructure *η*_Fe2O3_ = −6.8%/(10^7 ^A cm^-2^) is more than five times higher than the benchmark ferrimagnetic yttrium iron garnet (YIG) with *η*_YIG_ ~ −1.1%/(10^7 ^A cm^-2^)^22^. The magnetic field dependence of *f*_m_ in the same *α*-Fe_2_O_3_ sample is also measured. No clear change in *f*_m_ is observed up to 0.2 T magnetic field (Supplementary Note 13). This further supports the effectiveness of the SOT-tuning in *α*-Fe_2_O_3_ magnon. Although other stimuli such as strain^46^, Joule heating^23,47^ and magnetic fields^3^ can potentially realise a similar or even greater tunability, the application scenarios are severely limited by their substantial energy consumption and poor device scalability. SOT-tuning requires only a nanometre-thick spin source, which is a critical feature for the miniaturisation of future spintronic devices.

Our work provides an efficient and universal means to manipulate sub-THz magnons electrically in antiferromagnets. The SOT manipulation of magnons is applicable to a wide range of AFM materials without utilising exotic spin textures or complicated device structures. It, therefore, can play a significant role in developing all-electrical THz magnonic and photonic devices. For example, it can be used to create on-chip integrated THz photonics nano-circuits^48^.

## Methods

### (0001) cut *α*-Fe_2_O_3_ single crystal

The *α*-Fe_2_O_3_ single-crystalline sample with a thickness of 1 mm was purchased from MaTecK Material-Technologie & Kristalle GmbH (Germany). The sample surface was polished with a surface roughness of about 1 nm and a miscut orientation angle below 0.1°.

### Device fabrication

Prior to device fabrication, the *α*-Fe_2_O_3_ sample surface was cleaned with acetone, isopropyl alcohol (IPA) and deionised (DI) water by a standard ultrasonic bath process. A maskless ultraviolet lithography system (TTT-07-UVlitho, TuoTuo Technology (Singapore) Pte. Ltd) was used for device patterning with channel dimensions of 4 μm wide and 8 μm long for subsequent deposition (inset of Fig. 1d). The Pt layer was deposited on *α*-Fe_2_O_3_ with a thickness of 5 nm using DC magnetron sputtering at a base pressure of 9 × 10^-9 ^Torr. A 4 nm-thick SiO_2_ insulating layer was deposited to protect the device from oxidation.

### Spin Hall magnetoresistance measurements

In response to an external field, the orientations of magnetic order in *α*-Fe_2_O_3_ are coupled with spin currents injected from the adjacent Pt layer via the spin Hall effect, manifesting the spin Hall magnetoresistance (SMR)^49^. The AC longitudinal harmonic signals for Hall bar devices were measured in a physical property measurement system (PPMS, Quantum Design) at room temperature. During the measurement, a constant amplitude sinusoidal current with a frequency of 13.7 Hz was applied to the devices by a Keithley 6221 current source. The angle-dependent first-harmonic longitudinal voltage *V*_f_ was recorded by a Stanford research SR830 lock-in amplifier to obtain the first-harmonic longitudinal resistance *R*_f_ while rotating the in-plane magnetic field of 1 T. The negative sign of SMR with a value of Δ*R*/*R* ~ 0.2 % indicates an excellent interfacial quality of spin injection into *α*-Fe_2_O_3_ (Supplementary Note 14).

### Time-resolved magneto-optical measurements

In the time-resolved magneto-optical setup, the probe beam was the output of an 80 MHz Ti: sapphire femtosecond laser system (Spectra-Physics Mai Tai, pulse duration of 75 fs) centred at 1.55 eV (800 nm), and the pump beam was the second harmonic by using a *β*-Barium borate single crystal to 3.1 eV (400 nm). Both pump and probe pulses were linearly polarised. The fluence ratio between the pump and probe beams was around 10:1. Both beams were focused onto the sample surface at normal incidence by a ×50 objective lens (the pump beam diameter is ~1.8 μm with a typical fluence of 1.0 mJ cm^-2^; the probe beam diameter is ~1.2 μm with a typical fluence of 100 μJ cm^-2^). The magnon detection depth is the order of the penetration length of probe beams in *α*-Fe_2_O_3_, which is around 300 nm^50^. In this case, the reduced amplitude of SOT away from the α-Fe_2_O_3_/Pt interface potentially leads to the broadening of linewidth and reduced amplitude of magnon spectra in the magneto-optical results (Supplementary Note 15). A continuous flow cryostat system (ST-300, Janis) was used to set the sample temperature between 77 and 300 K with fluctuations below 0.1 K. For the applied current, square-wave-liked current pulses (a pulse amplitude: 0-4 mA; a pulse width: 20 μs; duty cycle: 2%) were generated by using a current source (Keithley 6221) and synchronised with the femtosecond laser beams through a function generator (DS345, Stanford Research). Reflectivity and the polarisation rotation of the probe beams were measured by using a Wollaston prism and a balanced detector combination (Supplementary Fig. 1). To be noted, the pure magnetic linear birefringence cannot be demodulated by using an HWP. However, in cases where both magnetic linear birefringence and dichroism simultaneously manifest, both the polarisation and ellipticity of the probe beam become subject to tuning. In such circumstances, the oscillating signal emanating from magnons can be detected through the utilisation of either an HWP or by strategically manipulating the optical axis of a Wollaston prism.

In order to excite *α*-Fe_2_O_3_ magnons efficiently, a 400 nm (3.1 eV) femtosecond pulse is used for pumping. The optical radiation with photon energy larger than the bandgap (2.14 eV)^50^ of *α*-Fe_2_O_3_ induces the strong electronic transition from O^2-^ to Fe^3+^ ions^51^ (Supplementary Note 2). The spin excitation, mediated by the intense electronic transition, is strongly confined near the surface due to a finite penetration depth (~29 nm) of the pump light. Therefore, the spatially non-uniform and transient excitation ignites broadband magnons^40^. The oscillation signal of the manipulated magnons is detected by the polarisation rotation of the probe beam via the magnetic-optical interactions. It occurs due to the different refractive indices of the probe between the one parallel to the **l** and the other perpendicular to it on the *α*-Fe_2_O_3_ (0001) plane, which shows a sinusoidal signal as a function of the polarisation angle of probe light (Supplementary Fig. 4b). The limitation of the measurement scheme is that the thermal effect disturbs the magnon signal at higher *J*_c_ > 2 × 10^7 ^A cm^-2^, which prevents from conducting any meaningful analysis (details are shown in Supplementary Note 16). Therefore, we restrict the data analysis to *J*_c_ < 2 × 10^7 ^A cm^-2^ in this work.

### Magnetic domain measurement

At room temperature, we measure the magnetic domain of the α-Fe_2_O_3_(0001) single crystal using magneto-optical Kerr microscopy. Longitudinal geometry is employed to probe the canted in-plane magnetic moment of *α*-Fe_2_O_3_(0001). Similar to the earlier report^8^, we observe distinct magnetic domains with a domain size over hundreds of micrometres (Supplementary Note 17). As this domain size much exceeds the size of SOT-tuned devices, it is reasonable to consider the main results under mono-domain conditions.

### Atomistic spin simulations for *α*-Fe_2_O_3_ magnon

We construct an atomistic Landau-Lifshitz-Gilbert equation, including the damping-like SOT

2 $$\partial {{{{{{\bf{m}}}}}}}_{{{{{{\rm{i}}}}}}}/\partial t=-\gamma {{{{{{\bf{m}}}}}}}_{{{{{{\rm{i}}}}}}}\times {{{{{{\bf{H}}}}}}}_{{{{{{\rm{eff}}}}}},{{{{{\rm{i}}}}}}}+\beta {{{{{{\bf{m}}}}}}}_{{{{{{\rm{i}}}}}}}\times \partial {{{{{{\bf{m}}}}}}}_{{{{{{\rm{i}}}}}}}/\partial t+{J}_{{{{{{\rm{m}}}}}},{{{{{\rm{z}}}}}}}{{{{{{\bf{m}}}}}}}_{{{{{{\rm{i}}}}}}}\times ({{{{{{\bf{m}}}}}}}_{{{{{{\rm{i}}}}}}}\times {{{{{\boldsymbol{\sigma }}}}}}),$$

where ${{{{{{\bf{m}}}}}}}_{{{{{{\rm{i}}}}}}}$ is the unit vector of magnetisation at site *i*, **H**_eff,i_ is the effective field, and ***σ*** is the spin polarisation. We use the following parameters of *α*-Fe_2_O_3_ at room temperature: the exchange field *H*_E_ = 930 T, the hard-axis anisotropy field *H*_Kz_ = –16.8 mT, the basal anisotropy field *H*_B_ = 1 μT, which originates from spontaneous magnetostriction^52^, the Dzyaloshinskii-Moriya field *H*_D_ = 2.5 T, and *γ* = 1.76 × 10^11 ^T s^-1^. *J*_m,z_ is the magnon spin current injected from Pt (Supplementary Note 9). To verify the dispersion relation, we apply an external ac magnetic field *H*_ext_ = 10 mT × sinc(2*πft*) and spatially distribute the magnetic field *H*_ext_ = 0.1 mT × sinc(*k*(*z-z*_0_)) for the ignition of broadband magnons where *z*_0_ is the surface of *α*-Fe_2_O_3_ and magnons propagate along the *z* direction. The DC magnon spin current is applied to the entire AFM system with the polarisation along the *x* and *y* directions. Numerical simulations were conducted from 0 to 1 μs with a minimum time interval of 0.1 ps with 10,000 spins. Magnon spin current is applied over a distance from 0 to 150 nm. The same parameters are used in theoretical and numerical calculations.

### Supplementary information

Supplementary Information

Peer Review File

## Data Availability

All other data that support the plots within this paper and other findings of this study are available from the Supplementary Note or the corresponding author upon reasonable request.

## Code availability

The code that has been used for this work is available from the corresponding author upon reasonable request.
