# Supplementary material for: Spin-orbit torque manipulation of sub-terahertz magnons in antiferromagnetic α-Fe2O3
Source: Nat Commun. 2024 May 14;15:4046. doi: 10.1038/s41467-024-48431-w (PMC11094109; doi:10.1038/s41467-024-48431-w)
Supplement: Supplementary file 1 — Supplementary Information [file 41467_2024_48431_MOESM1_ESM.pdf]

Supplementary Information

Spin-orbit torque manipulation of sub-terahertz magnons in antiferromagnetic α-Fe_2O_3

Dongsheng Yang[1†], Taeheon Kim[1,2†], Kyusup Lee[1], Chang Xu[1], Yakun Liu[1], Fei Wang[1], Shishun Zhao[1], Dushyant Kumar[1] and Hyunsoo Yang[1*]

Correspondence author. E-mail: eleyang@nus.edu.sg

Note 1: The spin dynamics of antiferromagnetic α-Fe_2O_3 at room temperature

As shown in Fig. 2b of the main text, the Fe^{3+} spins in α-Fe_2O_3 are ordered antiferromagnetically with a slight canting angle due to the Dzyaloshinskii-Moriya (DM) interaction. We consider the spin dynamics of α-Fe_2O_3 in a one-dimensional space along the z direction. We introduce the antiferromagnetic (AFM) order $\mathbf{l} = (\mathbf{m}_1 - \mathbf{m}_2)/2$ and ferromagnetic order $\mathbf{m} = (\mathbf{m}_1 + \mathbf{m}_2)/2$, where $\mathbf{m}_{1,2}$ is the sublattice magnetization. In a continuum medium, the continuously varying spin structure in space is expressed as $\mathbf{l}' = \frac{d\mathbf{l}}{dz} \sim \frac{\mathbf{l}_{i+1} - \mathbf{l}_i}{\Delta}$, where Δ is the unit length between neighbour lattice sites i+1 and i. A theoretical description of DM interaction for a canted AFM phase of α-Fe_2O_3 is described as $F_{\mathrm{DMI}} = \int D\hat{\mathbf{z}} \cdot (\mathbf{l} \times \mathbf{l}') + D\hat{\mathbf{z}} \cdot (\mathbf{m} \times \mathbf{l}) dz$ where the first term and second term are called inhomogeneous and homogeneous DM interaction, respectively. Above T_M, the canted AFM phase allows the second term. Therefore, the total energy density F for the canted antiferromagnet is

$$F = a/2|\mathbf{m}|^2 + A/2|\mathbf{l}'|^2 - K_z/2(\mathbf{l}\cdot\hat{\mathbf{z}})^2 - K_y/2(\mathbf{l}\cdot\hat{\mathbf{y}})^2 + \mathbf{D}\cdot(\mathbf{m}\times\mathbf{l}), \quad \text{(S1)}$$

where a and A are the homogeneous and inhomogeneous exchange constant, respectively[1]. The parameters are defined as $A = \Delta_a^2 \hbar\omega_E$, $a = 2\hbar\omega_E$, and $\mathbf{D} = \hbar\omega_D \mathbf{e}_y$ and Δ_a is set to the length between neighbours along the z axis. K_z and K_y are the uniaxial anisotropy along the z and y direction, respectively. Using the effective fields, $\mathbf{h}_l = -\partial F/(M_s \partial \mathbf{l})$ and $\mathbf{h}_m = -\partial F/(M_s \partial \mathbf{m})$, the coupled equation of motion for α-Fe_2O_3 is

$$\dot{\mathbf{l}} = (\gamma \mathbf{h}_m - \beta \dot{\mathbf{m}}) \times \mathbf{l}, \quad \text{(S2a)}$$

$$\dot{\mathbf{m}} = (\gamma \mathbf{h}_l - \beta \dot{\mathbf{l}}) \times \mathbf{l}, \quad \text{(S2b)}$$

where γ is the gyromagnetic ratio and β is magnetic damping. Taking the cross product of $\mathbf{l}$ in Eq. S2a and neglecting the high-order elements, the magnetization takes the form of

$$\mathbf{m} \sim \frac{1}{a}(\dot{\mathbf{l}} \times \mathbf{l} - \mathbf{D} \times \mathbf{l}). \quad \text{(S3)}$$

By inserting Eq. S3 to Eq. S2b, the dynamical equation of motion regarding **l** is given by

$$a\gamma[-\ddot{\mathbf{l}}/(a\gamma)+A^2\mathbf{l}''+K_z l_z\hat{\mathbf{z}}+K_y l_y\hat{\mathbf{y}}+\gamma/a\mathbf{D}(\mathbf{l}\cdot\mathbf{D})-\beta\dot{\mathbf{l}}/\gamma]\times\mathbf{l}-\gamma\mathbf{D}\times\dot{\mathbf{l}}=0. \quad \text{(S4)}$$

At room temperature, the magnetic ground state of α-Fe_2O_3 is $\mathbf{l} = \mathbf{e}_y$ and $\mathbf{m} = \mathbf{e}_z H_D/(2H_E)$, where $H_E = \omega_E/\gamma$ is the exchange field, $H_D = \omega_D/\gamma$ is the DM field. Two eigenmodes of spin oscillation regarding $\theta(t)$ and $\varphi(t)$ are derived with ansatz $\boldsymbol{l}=\{l_x, l_y, l_z\}=\{-\cos\theta\sin\varphi,\cos\theta\cos\varphi,-\sin\theta\}$:

$$\ddot{\theta}+2\omega_E\beta\dot{\theta}+(\omega_m^{qAFM,k=0})^2\theta=0, \quad \text{(S5a)}$$

$$\ddot{\varphi}+2\omega_E\beta\dot{\varphi}+(\omega_m^{qFM,k=0})^2\varphi=0. \quad \text{(S5b)}$$

Here, $f_m^{qAFM,k=0}=\frac{\omega_m^{qAFM,k=0}}{2\pi}=\frac{\gamma}{2\pi}\left(H_D^2+2(H_{Ky}-H_{Kz})H_E\right)^{1/2}$ and $f_m^{qFM,k=0}=\frac{\gamma}{2\pi}\left(2H_{Ky}H_E\right)^{1/2}$ are the quasi-antiferromagnetic (q-AFM) and quasi-ferromagnetic (q-FM) resonance modes at $k = 0$ respectively, ignoring the damping β, with the anisotropic field $H_{Kz}=\omega_{Kz}/\gamma$ ($H_{Ky}=\omega_{Ky}/\gamma$). The q-AFM resonance mode hosts dominated oscillations of $\mathbf{l}_z$ and $\mathbf{m}_x$ with sub-THz (~200 GHz) frequencies at room temperature, schematically illustrated in Fig. 2b in the main text. These time-dependent oscillating motions of **l** and **m** can be tracked using a time-resolved magneto-optical detection scheme.

Note 2: Laser-driven spin excitation of antiferromagnetic α-Fe_2O_3 at room temperature

The illustration of time-resolved magneto-optical measurement is shown in Supplementary Figure 1. Femtosecond laser pulses with a finite optical spectrum are conventionally regarded as an instantaneous stimulant to the collective AFM spin dynamics. This is because the timescale of laser pulse width (~0.1 ps) is one order of magnitude shorter than that of the AFM spin dynamic period (1~10 ps). In our time-resolved magneto-optical measurement, the photon energy of the pump light (3.1 eV)

exceeds the optical bandgap of α-Fe_2O_3 (E_g = 2.14 eV)[2]. As a result, a charge-transfer electronic transition occurs between the $2p$ nonbonding orbitals ($6t_{1u}$,$1t_{1u}$) of the oxygen site and the lowest energy orbital ($2t_{2g}\downarrow$) of the neighbouring iron site. Such a transition materializes within a typical timescale of 0.5 ps (as evidenced by the rise time of the time-solved magneto-optical signal in Supplementary Figure 2)[2]. It manifests the microscopic magnetic interactions (exchange interaction[3], DM interaction[4], magnetic anisotropy[5] and *d*-*f* exchange[6]) through a so-called magnetic-refraction process. These processes are particularly significant in the strongly correlated α-Fe_2O_3 since these interactions share the same electrostatic origin of Coulomb repulsion, which is strongly manifested by its electronic distributions[7].

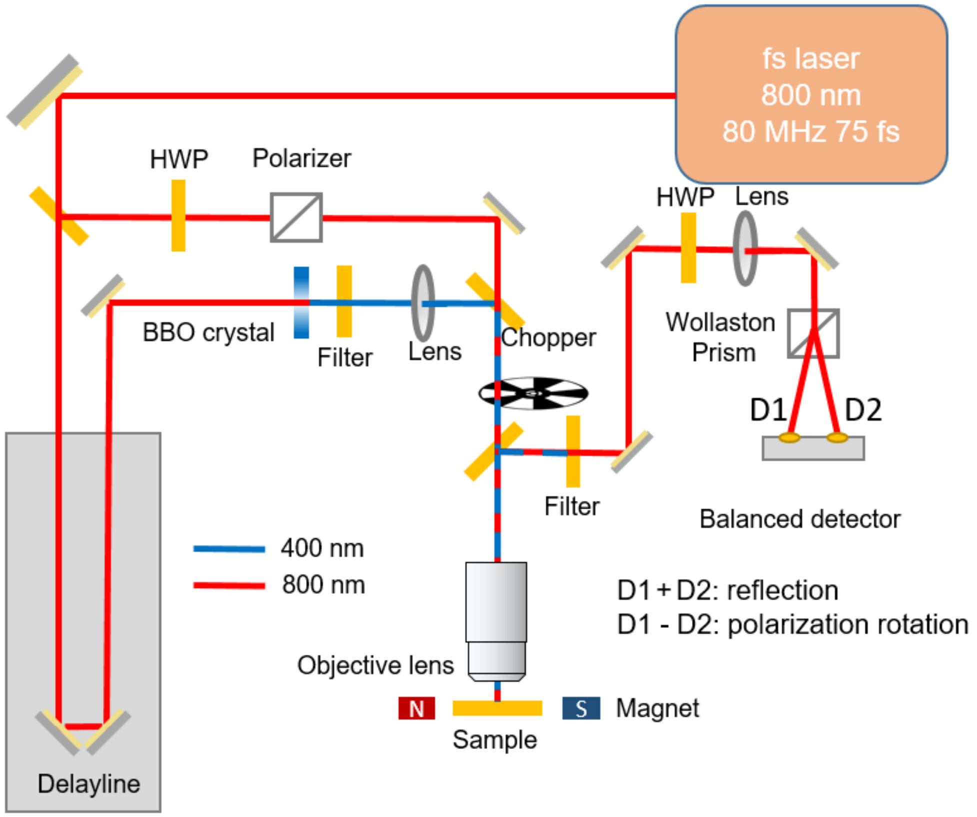

Supplementary Figure 1 | Time-resolved magneto-optical measurement. D1 and D2 indicate the detection channels of a balanced photodiode. The sum (V_{D1} + V_{D2}) and difference (V_{D1} − V_{D2}) of balanced photodiode signals indicate the reflectivity and polarisation rotation, respectively. The probe incident angle is 0°. HWP: half-wave plate. BBO: β-BaB_2O_4.

Compared to other magneto-optical-based spin excitation phenomena, such as the inverse Faraday effect and inverse Cotton-Mouton effect, the characteristic feature of the magnetic-refraction process is isotropic to the polarization of pump light. This has been confirmed in Supplementary Figure 3. Similar to previous studies on another AFM insulator $DyFeO_3$[8], this laser-triggered spin dynamics starts at a finite spin deflection on the sample surface determined by the laser profile and then proceed to propagate along the thickness direction. Comparatively, similar spin dynamics can be also launched through light-driven infrared-active phonons, but with a relatively longer timescale (~10 ps) as reported previously[7].

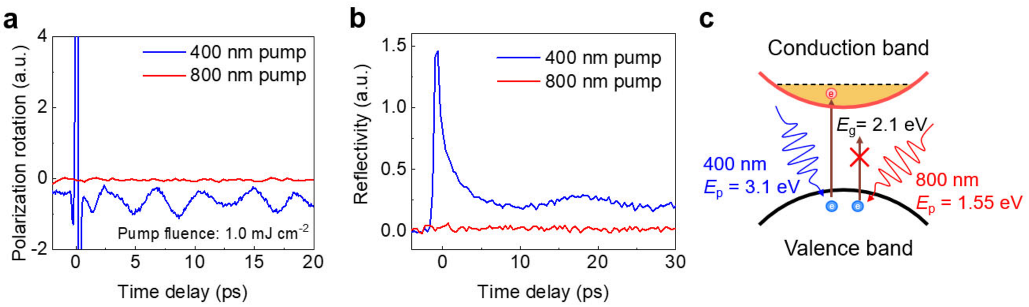

Supplementary Figure 2 | Pump wavelength-dependence on magnon and phonon excitation. a, Time-resolved magneto-optical result of α-Fe_2O_3(0001) using different pump energies of E_p = 3.1 eV (400 nm) and 1.55 eV (800 nm). The phonon baseline has been removed to highlight the magnetic component of the dynamics. The pump fluence is identical to 1 mJ cm^{-2} for both pump photon energies. **b**, The corresponding reflectivity result of α-Fe_2O_3. **c**, Schematics of the laser-induced charge-transfer electronic transition in α-Fe_2O_3 by the pump pulse. The spin dynamics is mediated with the charge-transfer transitions from O^{2-} to Fe^{3+} ions in α-Fe_2O_3 using a pump pulse with an energy higher than the optical band gap (~2.14 eV) of α-Fe_2O_3.

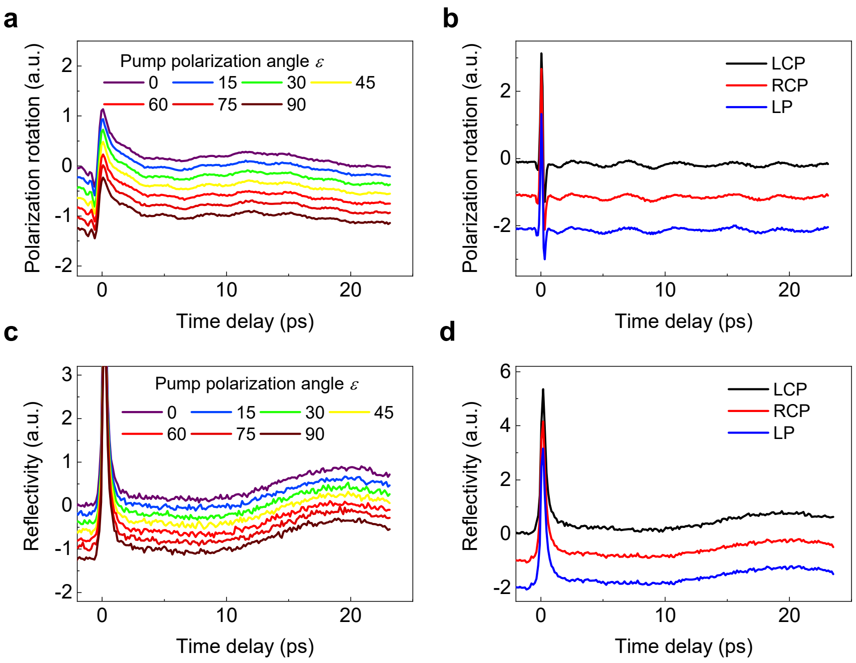

Supplementary Figure 3 | Pump polarisation and helicity-dependence on magnon and phonon excitation. a,**b**, The time-resolved magneto-optical measurement results under different pump polarisations (**a**) and helicities (**b**). The pump polarisation angle $\varepsilon = 0$ indicates a horizontal polarisation. LP, LCP, and RCP refer to linearly polarised, left-handed circularly polarised, and right-handed circularly polarised light, respectively. The vertical offsets are applied for clarity, and no difference is observed under various pumping polarisations. **c**,**d**, The corresponding time-resolved reflectivity measurement results under different pump polarisations (**c**) and helicities (**d**). It is noted that the phonon oscillations are overlapped on the time-resolved magneto-optical results, but the magnon oscillations are not seen in the time-resolved reflectivity results.

Note 3: Magneto-optical detection of α-Fe_2O_3 magnons

Typically, first-order magnetic-optical effects (Kerr effect and Faraday effect) are not useful to directly probe the AFM spin dynamics due to the absence of net magnetic moment **m**, especially for thin-film samples. Alternatively, second-order magnetic-optical effects (Voigt effect or Cotton-Mouton effect) can be directly coupled with **l** and thus are an ideal mechanism for AFM spin dynamic studies[9]. At room temperature,

α-Fe_2O_3 has both finite **m** and **l** owing to the DM interaction. In order to distinguish the dominated detection mechanism, we measure the parity change of oscillation signals with the reversal of the external magnetic field. As shown in Supplementary Figure 4a, no parity change is observed under the reversal of the magnetic field. This even symmetry feature indicates the electric field of light **E** couples with **l** rather than **m**, showing that the second-order MO effect, or Voigt effect, is the dominant detection mechanism.

In addition, the transient Voigt rotation signal ΔP can be derived using the Jones Matrix $\Delta P \sim \left(V_{\mathrm{D1}} - V_{\mathrm{D2}}\right) / \left(V_{\mathrm{D1}} + V_{\mathrm{D2}}\right) \sim \sin\left(2\xi + \Delta\varphi\right)\delta\Gamma\left(t\right)$, where V_{D1} (V_{D2}) is the response of channel D1(2) of a balanced photodiode (Supplementary Figure 1), ξ is the angle between **E** of the probe light and AFM order **l**, $\Delta\varphi$ is the additional phase due to oblique incidence, and $\Gamma(t)$ is the time delay between the pump and probe light. As shown in Supplementary Figure 4b, the probe angle-dependence of spectra amplitude matches well the Voigt rotation equation where the sign reversal of oscillation signal occurs when **E** // **l** and two maximum points are observed at ± 45°. The finite offset $\Delta\varphi$ can originate from the reflection measurement geometry. Similar results have been reported in α-Fe_2O_3[9] and $FeBO_3$[10,11] that a tilted sample orientation with light polarization direction can lead to a non-zero off-diagonal dielectric constant ε_{xy}, inducing a rotation of the probe light polarization. Comparatively, the polarity of q-FM magnon mode reverses with the direction of the in-plane magnetic field, implying that its detection mechanism can be the Kerr effect instead (Supplementary Figure 5).

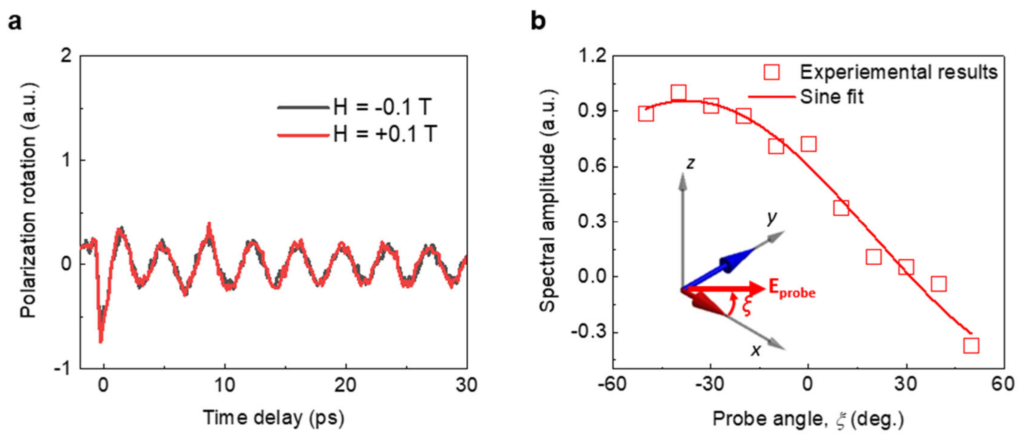

Supplementary Figure 4 | Magnetic field and probe polarisation dependence of q-AFM magnons. a, Time-resolved magneto-optical result of q-AFM magnons with 0.1 T in-plane positive (negative) fields. The low-frequency baseline has been removed to highlight the q-AFM dynamics. **b**, The spectral amplitude of the q-AFM magnon mode as a function of the probe polarisation angle ξ. The solid line corresponds to a sine fit with the probe angle.

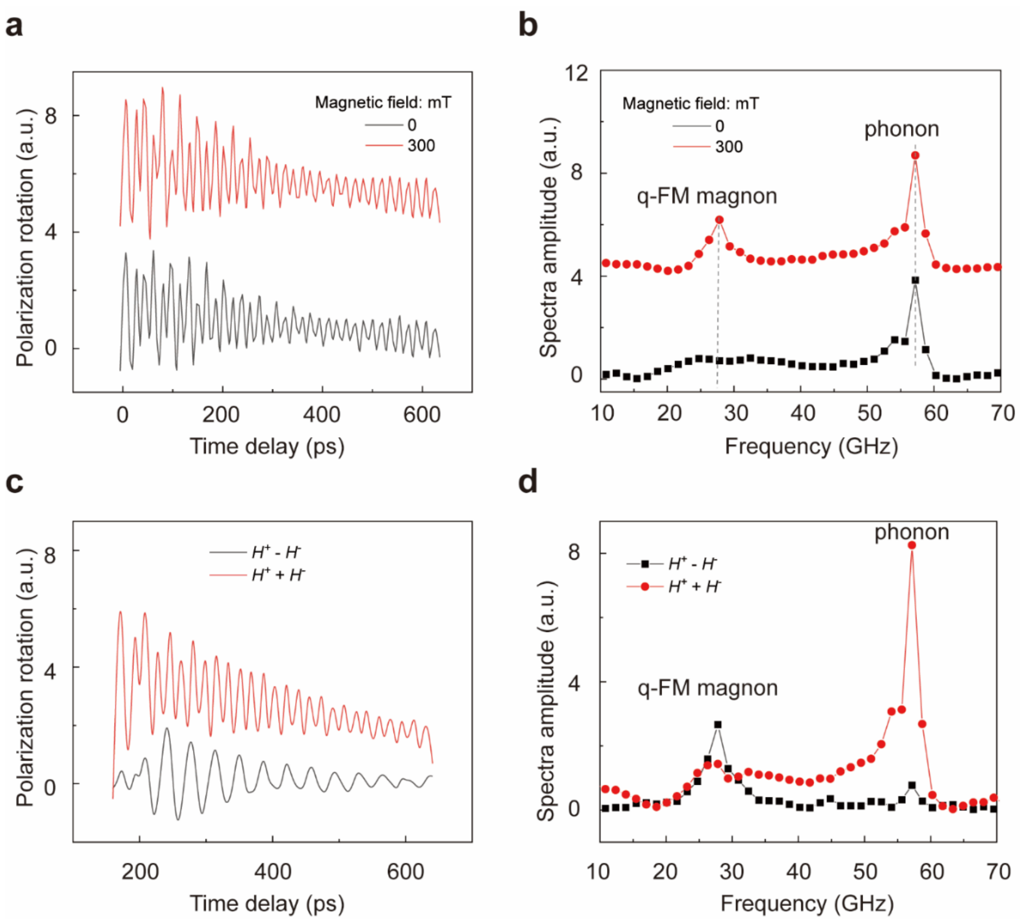

Supplementary Figure 5 | Symmetry analysis of q-FM magnon in α-Fe_2O_3 under the in-plane external magnetic field. **a**, Time-resolved magneto-optical measurement results with 0 and 300 mT in-plane magnetic fields. **b**, Corresponding magnon spectra by Fourier transform. The q-FM magnon is highly susceptible to the external magnetic field and can be observed only when the 300 mT in-plane magnetic field is applied. In contrast, the phonon mode remains invariant under external magnetic field conditions. **c**, The sum (H^+ + H^-) and difference (H^+ - H^-) of the oscillating signals. **d**, Corresponding FFT spectra. It shows that the phase of q-FM magnon oscillation is reversed with the opposite direction of the magnetic field while the phonon oscillation remains unchanged.

Note 4: The measurements of k = 0 magnons in α-Fe_2O_3

We used a conventional continuous-wave terahertz (cw-THz) spectroscopy setup to explore the spectral response of α-Fe_2O_3 at $k = 0$. The THz laser is radiated from the photo-mixer by optical heterodyning, with the frequency strictly at the difference frequency of two tunable infrared lasers. The THz magnetic field polarized along the **m** direction excites the q-AFM mode at $k = 0$, and a THz detector detects the transmitted THz waves by changing the frequency from 50 to 280 GHz. During the experiment, the THz beam path was purged with dry air to minimize absorption of THz radiation by water vapour, and the humidity was kept below 10%. A continuous flow cryostat system (ST-300, Janis) was used to set the sample temperature between 77 and 300 K.

Note 5: Refractive index calculation of α-Fe_2O_3/Pt heterostructure

If the layer size is much shorter than the light wavelength, the complex optical constants (e.g. refractive index and dielectric constant) of the multi-layer structure can be estimated by using the effective medium approximation (EMA)[12]. For the α-Fe_2O_3/Pt heterostructure in this work, the effective dielectric constant is given as

$$\tilde{\varepsilon}=\frac{\varepsilon_{r,Pt}t_{Pt}+\varepsilon_{r,Fe_2O_3}t_{Fe_2O_3}+i\left(\varepsilon_{i,Pt}t_{Pt}+\varepsilon_{i,Fe_2O_3}t_{Fe_2O_3}\right)}{t_{Pt}+t_{Fe_2O_3}} \tag{S6}$$

where the ε_r and ε_i refer to the real and imaginary component of the medium dielectric constant, respectively, and t_{Pt} and t_{Fe2O3} refer to the layer thickness of Pt and α-Fe_2O_3 layer. To be noted, we adopt the half-wavelength approximation value ($\lambda_{probe}/2$ = 400 nm) for t_{Fe2O3} considering the reflection geometry of time-resolved measurements. The complex dielectric constant of Pt and α-Fe_2O_3 is $\tilde{\varepsilon}_{Pt}$ = -64.92 + 9.31i and $\tilde{\varepsilon}_{Fe_2O_3}$ = 6.76 + 0i, respectively[13,14]. The effective refractive index n and absorption coefficient α_0 of the α-Fe_2O_3/Pt heterostructure at a wavelength of 800 nm is therefore extracted to be n = 2.60 and α_0 = 0.08 by using Eq. S6 and the relation between dielectric constant and refractive index as $n^2 = \frac{1}{2}\left[\sqrt{(\varepsilon_r^2+\varepsilon_i^2)}+\varepsilon_r\right]$ and $\alpha_0^2 = \left[\sqrt{(\varepsilon_r^2+\varepsilon_i^2)}-\varepsilon_r\right]/2$.

Note 6: SOT-manipulated k = 0 magnons in α-Fe_2O_3

To model the spin-orbit torque (SOT)-manipulated effects on the α-Fe_2O_3 magnon, we introduce the SOT terms on **l** and **m** to Eq. S2, given that

$$\dot{\mathbf{l}} = (\gamma \mathbf{h}_{\mathrm{m}} - \beta \dot{\mathbf{m}}) \times \mathbf{l} + \mathbf{\Gamma}^{\mathrm{l}}_{\mathrm{SOT}}, \tag{S7a}$$

$$\dot{\mathbf{m}} = (\gamma \mathbf{h}_{\mathrm{l}} - \beta \dot{\mathbf{l}}) \times \mathbf{l} + \mathbf{\Gamma}^{\mathrm{m}}_{\mathrm{SOT}}, \tag{S7b}$$

where the damping-like SOT in terms of **m** and **l** is defined as $\mathbf{\Gamma}^{\mathrm{l}}_{\mathrm{SOT}} = \zeta \mathbf{l} \times (\mathbf{m} \times \boldsymbol{\sigma})$ and $\mathbf{\Gamma}^{\mathrm{m}}_{\mathrm{SOT}} = \zeta \mathbf{l} \times (\mathbf{l} \times \boldsymbol{\sigma})$ where $\zeta = \frac{\gamma \hbar \theta_{\mathrm{H}} J_{\mathrm{c}}}{2ed_{\mathrm{AF}} M_{\mathrm{s}}} \gamma_{\mathrm{m}}$ is the effective SOT strength, $\boldsymbol{\sigma}$ is the spin polarization, J_c is the charge current density, $\hbar$ is the Planck constant, θ_H is the spin Hall angle, e is the electron charge, M_s is saturation magnetization of sublattice and γ_m is the space-averaged spin-to-magnon conversion ratio (see Note 9 for details of magnon current propagation). The values of these parameters are given in Methods section. Similar to Eq. S4, the dynamical equation of motion regarding **l** with SOT term is given by

$$a\gamma[-\ddot{\mathbf{l}}/(a\gamma) + A^2\mathbf{l}'' + K_z l_z \hat{\mathbf{z}} + K_y l_y \hat{\mathbf{y}} + \gamma / a \mathbf{D}(\mathbf{l} \cdot \mathbf{D}) - \beta \dot{\mathbf{l}} / \gamma] \times \mathbf{l} - \gamma \mathbf{D} \times \dot{\mathbf{l}} = a\zeta (\mathbf{l} \times \boldsymbol{\sigma}) \times \mathbf{l}. \tag{S8}$$

Dynamical equation of motion for k = 0 magnons for $\boldsymbol{\sigma}$ // l

Damping-like SOT does not reorient the equilibrium orientation of **l** because the external torque is zero when **σ** // **l** is at room temperature, as written in Eq. S8. Instead, the spin dynamics are similar to a coupled pendulum, which originates from SOT. For the k = 0 mode, the linearly coupled oscillations regarding $\theta(t)$ and $\varphi(t)$ under SOT are derived with ansatz $\boldsymbol{l} = \{l_x, l_y, l_z\} = \{-\cos\theta \sin\varphi, \cos\theta\cos\varphi, -\sin\theta\}$:

$$\ddot{\theta} + 2\omega_{\mathrm{E}} \beta \dot{\theta} + (\omega_{\mathrm{m}}^{\mathrm{qAFM},k=0})^2 \theta + \kappa_{\theta\varphi} \varphi = 0, \tag{S9a}$$

$$\ddot{\varphi} + 2\omega_{\mathrm{E}} \beta \dot{\varphi} + (\omega_{\mathrm{m}}^{\mathrm{qFM},k=0})^2 \varphi - \kappa_{\theta\varphi} \theta = 0. \tag{S9b}$$

Here, the SOT field $H_{SOT} = J_c\zeta/\gamma$, and the SOT spring constant $\kappa_{\theta\varphi} = 2\gamma^2 H_E H_{SOT}$ are defined. By applying J_c, the characteristic frequencies ($f = \omega/(2\pi)$) or eigenfrequencies of the system for $k = 0$ magnons are obtained as $f_m^{qAFM,k=0}(J_c) = \frac{\gamma}{2\pi}\left(\frac{H_D^2}{2} + H_E(-H_{Kz} + 2H_{Ky} + \beta^2 H_E) + \frac{1}{2}\left(\left(H_D^2 - 2H_{Kz}H_E + 2H_E H_{SOT}\right)\left(H_D^2 - 2H_{Kz}H_E - 2H_E H_{SOT}\right)\right)^{1/2}\right)^{1/2}$

and

$f_m^{qFM,k=0}(J_c) = \frac{\gamma}{2\pi}\left(\frac{H_D^2}{2} + H_E(-H_{Kz} + 2H_{Ky} + \beta^2 H_E) - \frac{1}{2}\left(\left(H_D^2 - 2H_{Kz}H_E + 2H_E H_{SOT}\right)\left(H_D^2 - 2H_{Kz}H_E - 2H_E H_{SOT}\right)\right)^{1/2}\right)^{1/2}$.

$\kappa_{\theta\varphi}$ plays a role in transferring energy back and forth between $\theta(t)$ and $\varphi(t)$. As J_c increases, the frequency of $k = 0$ q-AFM becomes reduced, as shown in Fig. 3c. $\kappa_{\theta\varphi}$ is proportional to H_E, implying that a higher H_E transfers the energy efficiently between θ(t) and φ(t).

Dynamical equation of motion for k = 0 magnons for σ // m

For the case of **σ // m**, SOT does not work as a spring. Instead, SOT reorients the **l** from the equilibrium or y axis. In the q-AFM mode at $k = 0$, the dynamical equation of motion is derived with the ansatz $\mathbf{l} = \{l_x, l_y, l_z\} = \{0, \cos\Phi, \sin\Phi\}$

$$\ddot{\Phi} + 2\omega_E\beta\dot{\Phi} + (\omega_m^{qAFM,k=0})^2 \sin(2\Phi)/2 = \omega_E\zeta J_c, \tag{S10}$$

where $f_m^{qAFM,k=0} = \frac{\gamma}{2\pi}\sqrt{H_D{}^2 - 2(H_{Kz} - H_{Ky})H_E}$. As J_c increases, the angle of **l** changes by Φ_0. The rotation of the reference axis by $-\Phi_0$ makes Eq. S10 recast as

$$\ddot{\theta} + 2\omega_E\beta\dot{\theta} + (\omega_m^{qAFM,k=0})^2 \cos(2\Phi_0)\sin(2\theta)/2 = -4\omega_E\zeta J_c \sin^2(\theta) \tag{S11}$$

where $\Phi(t) = \Phi_0 + \theta(t)$. By applying the current, the q-AFM frequency at $k = 0$ is modulated as $f_m^{qAFM,k=0}(\cos 2\Phi_0)^{1/2}$. The modulation factor $(\cos 2\Phi_0)^{1/2}$ originates from an anharmonic component of sin(2Φ) in Eq. S10, and is defined as $\cos 2\Phi_0 = \left(1 - \left(\frac{2f_{SOT}}{(f_m^{qAFM,k=0})^2/f_E}\right)^2\right)^{1/2} = \left(1 - \left(\frac{2H_{SOT}}{H_{eff,K}}\right)^2\right)^{1/2}$, where $f_m^{qAFM,k=0} = \frac{\gamma}{2\pi}(H_E H_{eff,K})^{1/2}$ and $H_{eff,K} = (2\pi f_m^{qAFM,k=0})^2/\gamma^2 H_E$.

Note 7: Spin-orbit torque manipulated magnon dispersion in α-Fe_2O_3

Magnon spectra for σ // l

According to Supplementary Note 6, the damping-like SOT does not reorient the **l**; thus, **l** remains aligned along the y axis. Therefore, initial ansatzes of small perturbation are set as $\delta l_\theta = A_y e^{i\omega t - ikz}$ and $\delta l_\varphi = A_x e^{i\omega t - ikz}$. The magnon dispersion relation and the group velocity of α-Fe_2O_3 magnon are given by

$$f_m(k) = \frac{\gamma}{2\pi}\left((\frac{a_0 H_E k}{2})^2 + \left(\frac{2\pi f_m(0)}{\gamma}\right)^2 \right)^{1/2}, \tag{S12a}$$

$$\upsilon_g(k) = \frac{\gamma}{2\pi}\frac{a_0^2 H_E^2 k}{\left((a_0 H_E k)^2 + \left(\frac{4\pi f_m(0)}{\gamma}\right)^2 \right)^{1/2}}. \tag{S12b}$$

Magnon spectra for σ // m

According to Supplementary Note 6, by applying the damping-like SOT, the angle of **l** changes by Φ_0. Since magnons are small fluctuations of **l** in an equilibrium state, it is easy to extract fluctuation around the equilibrium **l** using spherical coordinates. Now, it is converted into the new coordinates $\{\hat{r},\hat{\theta},\hat{\varphi}\} = \{1, A_\theta e^{i\omega t - ikr}, A_\varphi e^{i\omega t - ikr}\}$, and they are related to the Cartesian coordinates with the equilibrium phase Φ_0 shifted by the SOT field, H_{SOT}: $\hat{r} = \{0, -\sin\Phi_0, \cos\Phi_0\}$, $\hat{\theta} = \{0, -\cos\Phi_0, -\sin\Phi_0\}$ and $\hat{\varphi} = \{1,0,0\}$. Now, we can set magnon spectra from Eq. S4, by inserting a new ansatz with $\delta l_\theta = A_y e^{i\omega t - ikz}\cos\Phi_0 - A_z e^{i\omega t - ikz}\sin\Phi_0$ and $\delta l_\varphi = A_x e^{iwt - ikz}$. The magnon dispersion relation and group velocity take the forms:

$$f_m(k) = \frac{\gamma}{2\pi}\left((\frac{a_0 H_E k}{2})^2 + f_m^2(0)\cos(2\Phi_0) \right)^{1/2}, \tag{S13a}$$

$$\upsilon_g(k) = \frac{\gamma}{2\pi}\frac{a_0^2 \omega_E^2 k}{\left((a_0 H_E k)^2 + 4 f_m^2(0)\ \cos(2\Phi_0) \right)^{1/2}}, \tag{S13b}$$

where $\cos 2\Phi_0 = \left(1-\left(\frac{2f_{\mathrm{SOT}}}{f_0^2/f_{\mathrm{E}}}\right)^2\right)^{1/4} = \left(1-\left(\frac{2H_{\mathrm{SOT}}}{H_{\mathrm{eff,K}}}\right)^2\right)^{1/4}$. For example, at $J_c = 2 \times 10^7$ A cm^{-2}, f_m (0.0408) = 258.8 GHz where $H_{\mathrm{SOT}} = 2\pi J_c \zeta / \gamma$ = 404.7 Oe, Φ_0 = 1.2 rads (see Methods for parameters).

Note 8: Acoustic phonon mode in α-Fe_2O_3

As shown in Fig. 3b,c, two oscillation modes are excited by the 400 nm pump pulses in the time-resolved measurement. As discussed in the main text, the high-frequency component corresponds to the q-AFM magnon mode of α-Fe_2O_3. Meanwhile, the low-frequency component corresponds to the propagating acoustic phonon mode, which has been described in previous works[15,16]. Typically, this phonon mode is treated as a pump-induced strain wave propagating along the thickness direction. This strain wave modulates the refractive index of samples through optoacoustic interaction, which in turn modulates the light reflectivity observed in our time-resolved measurement. We confirmed the phonon origin of this 55 GHz oscillation through additional time-resolved reflectivity measurements (Supplementary Figure 3c,d), in which the oscillation at the same frequency manifests the light reflectivity.

Note 9: Drift-diffusion model and calculation for the magnon current profile in α-Fe_2O_3

We consider a Pt ($-t_{\mathrm{Pt}} < z < 0$)/α-Fe_2O_3 ($0 < z < t_{\alpha\mathrm{FO}}$) bilayer, where t_{Pt} (=5 nm) and $t_{\alpha\mathrm{FO}}$ (=1 mm) are the thickness of the Pt and α-Fe_2O_3 layer, respectively. In the Pt layer, the z-flowing spin current $\mathbf{J}_{s,z}$ is modeled[17] by

$$\mathbf{J}_{s,z}(z) = -\frac{\sigma_{\mathrm{Pt}}}{2e}\frac{\partial \mathbf{u}_s}{\partial z} - J_{\mathrm{SH}}\hat{\mathbf{y}} \quad \text{(S14)}$$

where σ_{Pt}, $J_{\mathrm{SH}}(=\zeta J_c)$, θ_{H}, $\mathbf{u}_s$ is the electrical conductivity, the spin Hall current density, and the spin chemical potential of the Pt layer, respectively. The effective SOT strength $\zeta = \frac{\gamma\hbar\theta_{\mathrm{H}}}{2ed_{\mathrm{AF}}M_s}$ is given by the reduced Planck constant $\hbar$ = 1.054 × 10^{-34} J s,

the spin Hall angle $\theta_H = 0.2$, the electron charge e, the effective penetration depth (d_{AF}) of spin current into α-Fe_2O_3 of ~0.5 nm, and the saturation magnetization for each of the sublattices of α-Fe_2O_3, $M_s = 715$ G. At the interface $z = 0$, $\mathbf{J}_{s,z}$ is governed by the spin accumulation and spin mixing conductance

$$\mathbf{J}_{s,z}(z=0)=\sum_{i=1.2} G_{re}\mathbf{m_i}\times(\mathbf{m_i}\times\mathbf{u_s})+G_{im}\mathbf{m_i}\times\mathbf{u_s} \tag{S15}$$

where i is the lattice site and G_{re} (G_{im}) is the real (imaginary) part of the effective spin mixing conductance. The diffusion equation is obtained by $\partial\mathbf{J_s}/\partial z = -\mathbf{u_s}/\tau$,

$$\frac{\partial^2\mathbf{u_s}}{\partial z^2}=\frac{\mathbf{u_s}}{\lambda_{pt}{}^2}, \tag{S16}$$

where λ_{Pt} is the spin diffusion length of the Pt layer. For $\boldsymbol{\sigma}$ // $\mathbf{m}$, $\mathbf{u}_s(0)$ at the interface is obtained by solving equations (S14)-(S16):

$$\mathbf{u}_s(0)=u_{s0}=-\hat{\mathbf{y}}\frac{2eJ_s\lambda_{pt}\tanh[\frac{t_{Pt}}{2\lambda_{pt}}]}{\sigma_{pt}+2G_{re}\lambda_{pt}\coth[\frac{t_{Pt}}{2\lambda_{pt}}]}. \tag{S17}$$

In α-Fe_2O_3 ($0 < z < t_{\alpha FO}$), the z-flowing magnon current density $\mathbf{J}_{m,z}$ is modeled[18] by

$$\mathbf{J}_{m,z}(z)=-\frac{\sigma_{\alpha FO}}{e}\frac{\partial\mathbf{u}_m}{\partial z}. \tag{S18}$$

The diffusion equation is obtained by

$$\frac{\partial^2\mathbf{u}_m}{\partial z^2}=\frac{\mathbf{u}_m}{\lambda_{\alpha FO}{}^2}, \tag{S19}$$

where $\mathbf{J}_{m,z}$, $\mathbf{u}_m$, $\tilde{\sigma}_{\alpha FO}$ and $\lambda_{\alpha FO}$ are the z-flowing magnon current, the magnon chemical potential, the magnon spin conductivity and the magnon diffusion length of the α-Fe_2O_3 layer, respectively. The interface spin current $J_s^{int} = g_s(u_s^{int} - u_m^{int})$ provides one boundary condition at the interface to the α-Fe_2O_3; where g_s is the effective spin conductance[18]. Another boundary condition is given by $J_{m,z}(d_{\alpha FO}) = 0$. Using equations (S17)-(S19), the magnon chemical potential and current profile along the **z** direction are shown in Supplementary Figure 6.

$\mathbf{J}_{m,z}$ becomes zero when z approaches $t_{\alpha FO}$. We plot $\mathbf{J}_{m,z}$ for the following parameters[19,20]: $\lambda_{\alpha FO}$ = 250 nm at room temperature, $\tilde{\sigma}_{\alpha FO}$ = 1 × 10^6 S m^{-1}, G_{re} = 10^{13} Ω^{-1} m^{-2}, d_{Pt} = 5 nm, λ_{Pt} = 3 nm, $\tilde{\sigma}_{Pt}$ = 6 × 10^5 S m^{-1} and g_s = ~10^{11} Ω^{-1} m^{-2}. For an observed magnon wavelength (~ 150 nm), $J_{m,z}$ profile is almost linear. Thereby, the space-averaged J_m modifies a spin-wave dispersion.

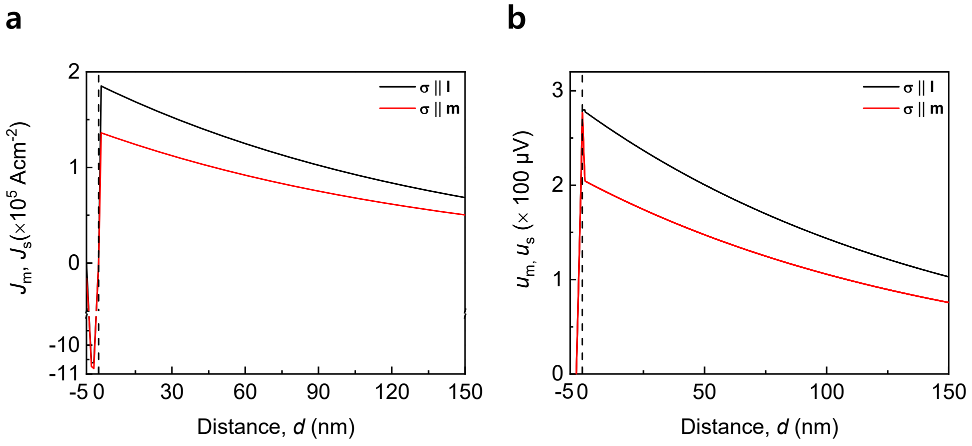

Supplementary Figure 6 | Magnon chemical potential (a) and current density (b) for J_c = 2 × 10^7 A cm^{-2} in α-Fe_2O_3. Distance (d) is the depth along the z direction from the α-Fe_2O_3/Pt interface.

Note 10: Simulation for current-induced heating in α-Fe_2O_3/Pt

We simulate the Joule heating with COMSOL Multiphysics to analyse the current-induced temperature change. Simulation parameters are from COMSOL Material Library and Ref. 21-23. Applying a 2 × 10^7 A cm^{-2} current pulse to the Pt layer for 20 μs, we investigate the temperature change of α-Fe_2O_3 at various depths and moments. As seen in Supplementary Figure 7, the Joule heating induces a temperature rise of up to 6 K at the centre α-Fe_2O_3/Pt interface during the probe laser detection. Considering the temperature-dependent f_m trend at ~300 K, the current-induced heating may lead to a slight increase of f_m. This result contrasts with our observed trend (f_m decreases with increasing J_c) in Fig. 3, therefore ruling out the possibility of current-induced heating effects as the primary origin of our observation.

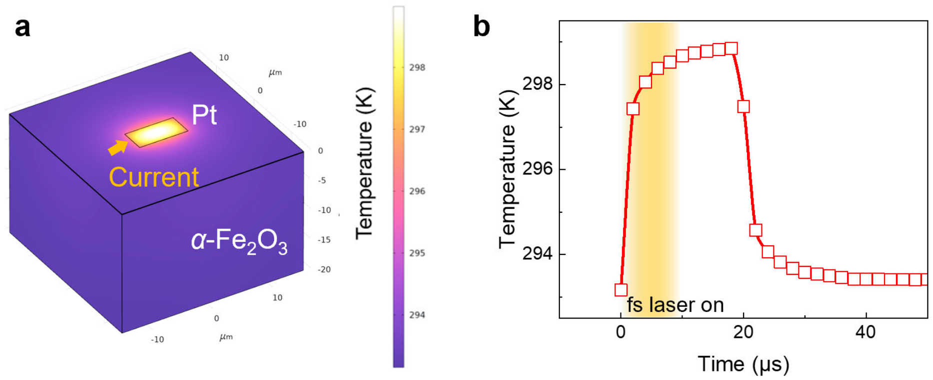

Supplementary Figure 7 | Simulation for current-induced heating effect. a, Simulation schematic of α-Fe_2O_3/Pt (5nm) devices. Current pulses are injected into the Pt layer and the associated Joule heating transfers from Pt to α-Fe_2O_3. **b**, Time-resolved temperature change at the centre position of the α-Fe_2O_3/Pt interface. The yellow-coloured region indicates the region with probe laser pulses. The estimated current-induced temperature change is 6 K, potentially leading to an increase of f_m. This is in contrast to the trend we observe in Fig. 3c (f_m decreases with J_c) of the main text, ruling out current-induced heating effects as a main mechanism of our observation.

Note 11: SOT effect on spin lifetime

As shown in Supplementary Figure 8, we did not observe a clear trend of SOT effect on the spin lifetime for both spin configurations as the applied current density is smaller than that for self-oscillation.

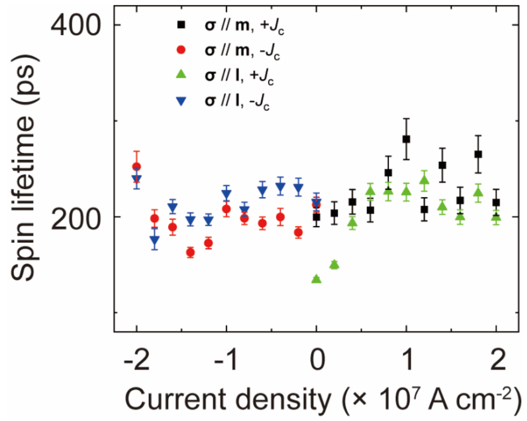

Supplementary Figure 8 | Current-dependent spin lifetime in α-Fe_2O_3.

Note 12: SOT effect of f_m tuning on different magnetic parameters for σ // m

Furthermore, we explore the SOT effect on the magnon frequency with different magnetic parameters in an insulating AFM by introducing exchange field H_E, effective anisotropy field $H_{eff,K}$ and the modulation factors χ_E and χ_K, where $H'_E = \chi_E H_E$, $H'_{eff,K} = \chi_K H_{eff,K}$ and $H_{eff,K} = f_m^{qAFM,k=0} / H_E$. As shown in Supplementary Figure 9, it concludes that η is proportional to H_E and $1/H_{eff,K}$ by considering that the η relies on how easily SOT reorients **l** for **σ** // **m**. In this case, AFMs with a high H_E and low $H_{eff,K}$ is preferred for efficient magnon frequency tuning.

The theoretical limit for the maximum frequency change by SOT is worth noting. For **σ** // **m**, the SOT compensates the effective anisotropy at $J_c^{th,l\perp\sigma} = \sim 3\times10^7$ A cm^{-2} (Supplementary Figure 9), above which the right angle oscillation around another axis occurs[24]. Therefore, $\Delta f = f_m(k, J_c^{th,l\perp\sigma}) - f_m(k, 0) = \sqrt{(\upsilon_0 k)^2 + f^2_{m,k=0}(J_c^{th,l\perp\sigma})} - \sqrt{(\upsilon_0 k)^2 + f^2_{m,k=0}(0)}$ = 210 – 272 GHz = – 62 GHz for $k = 4.08 \times 10^5$ cm^{-1} magnons. Since the thermal effect disturbs the detection of the magnon signal, we restrict our discussions below $J_c = 2 \times 10^7$ A cm^{-2} in this work in which the SOT effect is dominated.

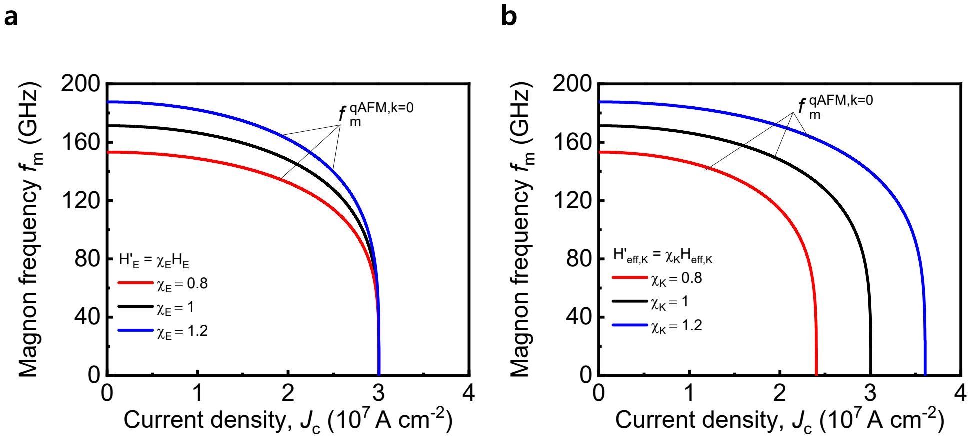

Supplementary Figure 9 | SOT induced frequency (f_m) modulation for k = 0 as a function of the exchange field H_E (a) and effective anisotropy $H_{eff,K}$ (b) at σ // m. The f_m tuning at a given current density J_c is more efficient in antiferromagnets with a higher H_E and a lower $H_{eff,K}$.

Note 13: Magnetic field-dependent f_m of α-Fe_2O_3 q-AFM magnon

To compare the effectiveness of f_m tuning in α-Fe_2O_3 by SOT, we investigate the magnetic field-dependence of f_m (Supplementary Figure 10). Our measurements indicate that no observable frequency change is observed within our measurement accuracy up to a magnetic field of 0.2 T, which supports our claim in the main text that the traditional magnetic field method is ineffective when dealing with antiferromagnets, similar to previous NiO results[25]. We utilize the Ampère's law for a rectangular plate to calculate the current-induced magnetic field of $H_{Oe} \sim \mu_0 J_C t_{Pt} / 2 = 0.63$ mT with $J_c = 2 \times 10^7$ A cm^{-2}, where μ_0 is the vacuum permeability and t_{Pt} is the thickness of the Pt layer. Based on the electron gyromagnetic ratio for typical ferromagnets, we estimate the upper limit of the frequency change induced by H_{Oe} to be $\gamma H_{Oe}/2\pi \approx 0.017$ GHz. It is worth noting that, even with this conservative estimation, the amount of the SOT-tuned f_m is 10^3 times larger than that by current-induced magnetic field H_{oe}. This result highlights a much greater efficiency of SOT over the static magnetic field in controlling the magnetic dynamics in AFM α-Fe_2O_3.

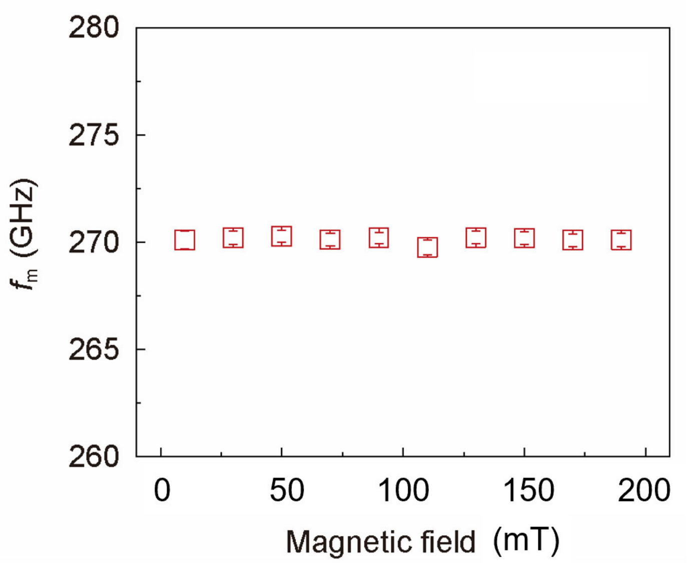

Supplementary Figure 10 | Magnetic field dependent q-AFM f_m in α-Fe_2O_3(0001). No clear f_m change is observed under the maximum magnetic field of up to 200 mT.

Note 14: Spin-Hall magnetoresistance

The large magnitude of magnetoresistance ~ 0.2 % in Supplementary Figure 14 indicates a decent magnetic interface between α-Fe_2O_3 and Pt.

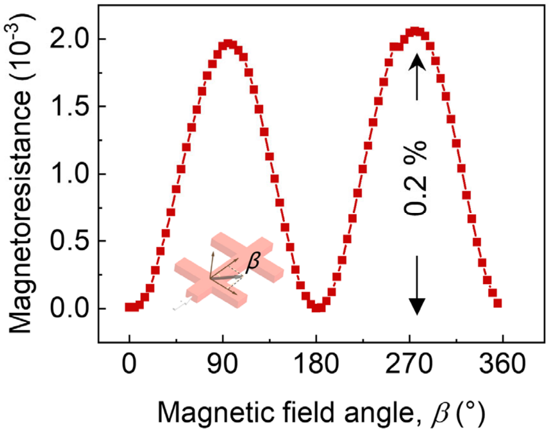

Supplementary Figure 11 | Spin-Hall magnetoresistance. Spin-Hall magnetoresistance is obtained by rotating the in-plane magnetic field with an angle β with respect to the long channel direction.

Note 15: α-Fe_2O_3 magnon spectra with current

As discussed in Note 9, the magnon detection distance of the magneto-optical method is comparable to that of the magnon decay length. In this case, the reduced amplitude of SOT away from the α-Fe_2O_3/Pt interface potentially leads to the broadening of linewidth and reduced amplitude of magnon spectra in the magneto-optical results. To confirm this, the magnon spectra in the frequency domain at $J_c = 0$ and 2×10^7 A cm^{-2} are shown in Supplementary Figure 12. The magnon amplitude decreases from 17.9 to 15.5 with increasing $J_c = 0$ to $2{\times}10^7$ A cm^{-2}. In the inset of Supplementary Figure 12, we observe a slight increase of normalised linewidth (df_m/f_m, df_m is the full width at half maximum of the magnon spectra) with increasing J_c.

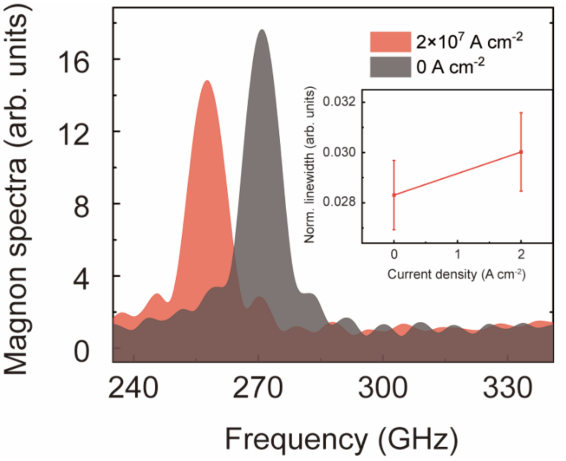

Supplementary Figure 12 | Magnon spectra at $J_c = 0$ and 2×10^7 A cm^{-2} in α-Fe_2O_3. The inset is the corresponding normalized linewidth.

Note 16: Current-manipulated f_m at higher j_c range

Supplementary Figure 13 demonstrates the magneto-optical measurement results of α-Fe_2O_3/Pt at higher J_c. As shown in the inset of Supplementary Figure 13, the magnon amplitude starts to drop with $J_c > 1.5 \times 10^7$ A cm^{-2}. This reduced magnon amplitude hinders our analysis for high J_c values.

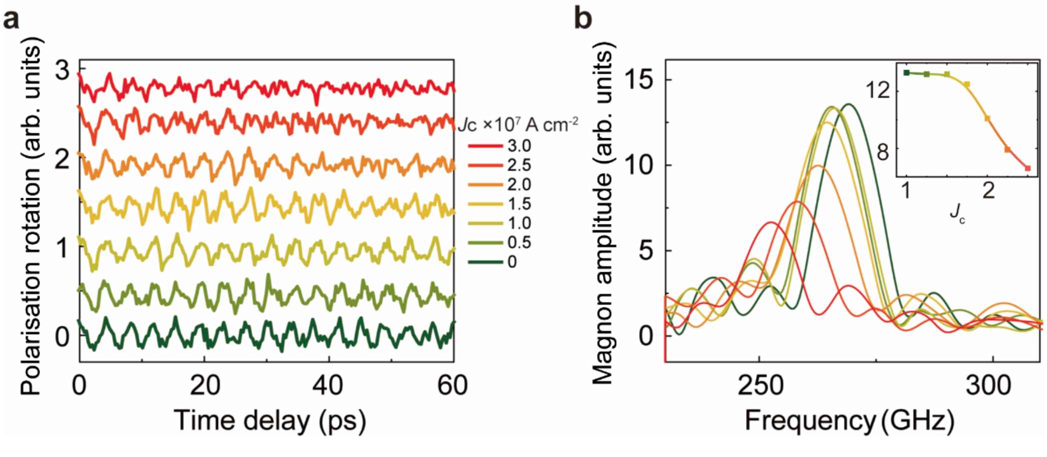

Supplementary Figure 13 | Current-dependent magnon amplitude in Fe_2O_3. a, Time-resolved polarization rotation results under various J_c. **b**, Corresponding magnon spectra. The magnon amplitude reduces with J_c, especially when $J_c > 1.5 \times 10^7$ A cm^{-2}. The inset indicates that peak magnon amplitude starts to decrease at $J_c > 1.5 \times 10^7$ A cm^{-2}.

Note 17: Magnetic domain image of α-Fe_2O_3(0001)

A uniform magnetic domain with a domain size of several hundred micrometres is observed in Supplementary Figure 13. The size is much larger than the size of SOT-tuned devices.

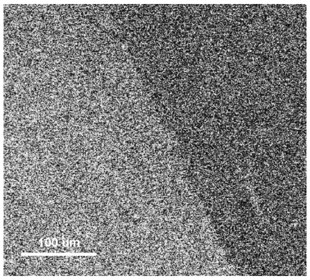

Supplementary Figure 14 | Magnetic domain image of α-Fe_2O_3(0001) single crystal measured by longitudinal MOKE microscopy at room temperature.

Supplementary Table 1 | Comparison of the electrical manipulated f_m. All other references deal with f_m tuning at $k = 0$, whereas our work considers $k = 0$ and $k = 4.08 \times 10^5$ cm^{-1} magnons. η: frequency tuning efficiency, H_0: the external magnetic field.

Material	f_m (GHz)	η (/10^7 A cm^{-2})	Tunability	Measurement condition	Driving force	Ref
CoFeB	1~4	-	+0.17 GHz/0.001 V	Room temp.	Voltage	26
NiFe	7	+1.4 %	+3 GHz/2.8×10^8 A cm^{-2}	Room temp.	Joule heating	27
CoGd	40-80	-7.5 %<br>+7.2 %	-20 GHz/6.8×10^7 A cm^{-2}<br>+40 GHz/6.8×10^7 A cm^{-2}	$T > T_C$ (Compensation temp. ~ 304 K)	Joule heating	28
YIG	3	-1.1 %	-0.4 GHz/1.25×10^8 A cm^{-2}	Room temp. H_0 ~ 0.065 T	SOT	29
CrI_3	70	-	-12.5 GHz/15 V	T ~ 4 K<br>H_0 ~ 0.5 T	Voltage	30
Fe_3Sn	6.6	-2.5 %	-0.15 GHz/2×10^7 A cm^{-2}	Room temp.	SOT	31
α-Fe_2O_3	272<br>(k=4.08×10^5 cm^{-1})<br>171<br>(k=0)	-2.6 ± 0.4 %<br>(k=4×10^5 cm^{-1})<br>-7 ± 1.0 %<br>(k=0)	-14 ± 2.0 GHz/2×10^7 A cm^{-2}<br>(k=4.08×10^5 cm^{-1})<br>-24 ± 3.1 GHz/2×10^7 A cm^{-2}<br>(k=0)	Room temp.	SOT<br>(**σ** // **m**)	This work

Reference for Supplementary Information

1. Hals, K. M., Tserkovnyak, Y. & Brataas, A. Phenomenology of current-induced dynamics in antiferromagnets. *Phys. Rev. Lett* **106**, 107206 (2011).
2. Marusak, L. A., Messier, R. & White, W. B. Optical absorption spectrum of hematite, α-Fe_2O_3 near IR to UV. *J. Phys. Chem. Solids* **41**, 981-984 (1980).
3. Mikhaylovskiy, R. V. *et al.* Resonant pumping of d-d crystal field electronic transitions as a mechanism of ultrafast optical control of the exchange interactions in iron oxides. *Phys. Rev. Lett.* **125**, 157201 (2020).
4. Mikhaylovskiy, R. V. *et al.* Ultrafast optical modification of exchange interactions in iron oxides. *Nat. Commun.* **6**, 8190 (2015).
5. Windsor, Y. W. *et al.* Deterministic control of an antiferromagnetic spin arrangement using ultrafast optical excitation. *Commun. Phys.* **3**, 139 (2020).
6. Subkhangulov, R. *et al.* All-optical manipulation and probing of the d–f exchange interaction in EuTe. *Sci. Rep.* **4**, 4368 (2014).
7. Afanasiev, D. *et al.* Ultrafast control of magnetic interactions via light-driven phonons. *Nat. Mater.* **20**, 607–611 (2021).
8. Hortensius, J. R. *et al.* Coherent spin-wave transport in an antiferromagnet. *Nat. Phys.* **17**, 1001-1006 (2021).
9. Grishunin, K., Mashkovich, E. A., Kimel, A. V., Balbashov, A. M. & Zvezdin, A. K. Excitation and detection of terahertz coherent spin waves in antiferromagnetic α–Fe_2O_3. *Phys. Rev. B* **104**, 024419 (2021).
10. Kalashnikova, A. *et al.* Impulsive generation of coherent magnons by linearly polarized light in the easy-plane antiferromagnet $FeBO_3$. *Phys. Rev. Lett.* **99**, 167205 (2007).
11. Mashkovich, E. *et al.* Terahertz optomagnetism: nonlinear THz excitation of GHz spin waves in antiferromagnetic $FeBO_3$. *Phys. Rev. Lett.* **123**, 157202 (2019).
12. Caligiuri, V., Pezzi, L., Veltri, A. & De Luca, A. Resonant gain singularities in 1D and 3D metal/dielectric multilayered nanostructures. *ACS nano* **11**, 1012-1025 (2017).
13. Werner, W. S., Glantschnig, K. & Ambrosch-Draxl, C. Optical constants and inelastic electron-scattering data for 17 elemental metals. *J. Phys. Chem. Ref. Data.* **38**, 1013-1092 (2009).
14. Querry, M. R. *Optical constants*. Report No. D-A158-623 (University of Missouri, 1985).
15. Takahara, M., Jinn, H., Wakabayashi, S., Moriyasu, T. & Kohmoto, T. Observation of coherent acoustic phonons and magnons in an antiferromagnet NiO. *Phys. Rev. B* **86**, 094301 (2012).
16. Hortensius, J. *et al.* Coherent spin-wave transport in an antiferromagnet. *Nat. Phys.* **17**, 1001-1006 (2021).
17. Marmion, S., Ali, M., McLaren, M., Williams, D. & Hickey, B. Temperature dependence of spin Hall magnetoresistance in thin YIG/Pt films. *Phys. Rev. B* **89**, 220404 (2014).
18. Cornelissen, L. J., Peters, K. J., Bauer, G. E., Duine, R. & van Wees, B. J. Magnon spin transport driven by the magnon chemical potential in a magnetic insulator. *Phys. Rev. B* **94**, 014412 (2016).
19. Lebrun, R. *et al.* Tunable long-distance spin transport in a crystalline antiferromagnetic iron oxide. *Nature* **561**, 222–225 (2018).

20 Han, J. *et al.* Birefringence-like spin transport via linearly polarized antiferromagnetic magnons. *Nat. Nanotechnol.* **15**, 563-568 (2020).

21 Severin, J. & Jund, P. Thermal conductivity calculation in anisotropic crystals by molecular dynamics: Application to $\alpha-Fe_2O_3$. *J. Chem. Phys.* **146**, 054505 (2017).

22 Kulikova, D. P. *et al.* Optical properties of tungsten trioxide, palladium, and platinum thin films for functional nanostructures engineering. *Opt. Express* **28**, 32049-32060 (2020).

23 Rakić, A. D., Djurišić, A. B., Elazar, J. M. & Majewski, M. L. Optical properties of metallic films for vertical-cavity optoelectronic devices. *Appl. Opt.* **37**, 5271-5283 (1998).

24 Lee, D. K., Park, B. G. & Lee, K. J. Antiferromagnetic oscillators driven by spin currents with arbitrary spin polarization directions. *Phys. Rev. Appl.* **11**, 054048 (2019).

25 Wang, Z. *et al.* Magnetic field dependence of antiferromagnetic resonance in NiO. *Appl. Phys. Lett.* **112**, 252404 (2018).

26 Kanai, S., Gajek, M., Worledge, D., Matsukura, F. & Ohno, H. Electric field-induced ferromagnetic resonance in a CoFeB/MgO magnetic tunnel junction under dc bias voltages. *Appl. Phys. Lett.* **105**, 242409 (2014).

27 Demidov, V. E. *et al.* Control of magnetic fluctuations by spin current. *Phys. Rev. Lett.* **107**, 107204 (2011).

28 Divinskiy, B., Chen, G., Urazhdin, S., Demokritov, S. O. & Demidov, V. E. Effects of spin-orbit torque on the ferromagnetic and exchange spin-wave modes in ferrimagnetic CoGd alloy. *Phys. Rev. Appl.* **14**, 044016 (2020).

29 Collet, M. *et al.* Generation of coherent spin-wave modes in yttrium iron garnet microdiscs by spin-orbit torque. *Nat. Commun.* **7**, 10377 (2016).

30 Zhang, X.-X. *et al.* Gate-tunable spin waves in antiferromagnetic atomic bilayers. *Nat. Mater.* **19**, 838-842 (2020).

31 Lyalin, I., Cheng, S. & Kawakami, R. K. Spin-orbit torque in bilayers of kagome ferromagnet Fe_3Sn_2 and Pt. *Nano Lett.* **21**, 6975-6982 (2021).
